# Neutrophil heterogeneity and plasticity: unveiling the multifaceted roles in health and disease

**DOI:** 10.1002/mco2.70063

**Published:** 2025-01-21

**Authors:** Weifeng He, Lingfeng Yan, Dongxue Hu, Jianlei Hao, Yih‐Cherng Liou, Gaoxing Luo

**Affiliations:** ^1^ Institute of Burn Research State Key Laboratory of Trauma and Chemical Poisoning the First Affiliated Hospital of Army Medical University (the Third Military Medical University) Chongqing China; ^2^ Chongqing Key Laboratory for Wound Repair and Tissue Regeneration Chongqing China; ^3^ Department of Biological Sciences Faculty of Science National University of Singapore Singapore Singapore; ^4^ Guangdong Provincial Key Laboratory of Tumor Interventional Diagnosis and Treatment Zhuhai Institute of Translational Medicine Zhuhai People's Hospital (Zhuhai Clinical Medical College of Jinan University) Jinan University Zhuhai Guangdong China; ^5^ The Biomedical Translational Research Institute Faculty of Medical Science Jinan University Guangzhou Guangdong China; ^6^ National University of Singapore (NUS) Graduate School for Integrative Sciences and Engineering National University of Singapore Singapore Singapore

**Keywords:** neutrophil functions, neutrophil heterogeneity, neutrophil plasticity, neutrophil signaling pathways, neutrophil‐targeted therapies

## Abstract

Neutrophils, the most abundant circulating leukocytes, have long been recognized as key players in innate immunity and inflammation. However, recent discoveries unveil their remarkable heterogeneity and plasticity, challenging the traditional view of neutrophils as a homogeneous population with a limited functional repertoire. Advances in single‐cell technologies and functional assays have revealed distinct neutrophil subsets with diverse phenotypes and functions and their ability to adapt to microenvironmental cues. This review provides a comprehensive overview of the multidimensional landscape of neutrophil heterogeneity, discussing the various axes along which diversity manifests, including maturation state, density, surface marker expression, and functional polarization. We highlight the molecular mechanisms underpinning neutrophil plasticity, focusing on the complex interplay of signaling pathways, transcriptional regulators, and epigenetic modifications that shape neutrophil responses. Furthermore, we explore the implications of neutrophil heterogeneity and plasticity in physiological processes and pathological conditions, including host defense, inflammation, tissue repair, and cancer. By integrating insights from cutting‐edge research, this review aims to provide a framework for understanding the multifaceted roles of neutrophils and their potential as therapeutic targets in a wide range of diseases.

## Graphical abstract

Neutrophils showed heterogeneity in multiple dimensions. Neutrophils exhibit different phenotypes under the influence of inflammatory, metabolic, environmental and other factors, and are controlled by multiple signaling pathways.

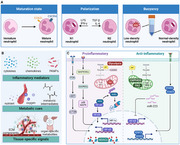

## 1 INTRODUCTION

Neutrophils, the most abundant circulating leukocytes, have long been recognized as the first line of defense against invading pathogens.^1^, ^2^ Traditionally viewed as short‐lived, terminally differentiated cells with a primary role in inflammatory response and antimicrobial defense, neutrophils have undergone a remarkable conceptual transformation over the past decade.^3^ Advances in single‐cell technologies and functional assays have unveiled the existence of distinct neutrophil subsets with diverse phenotypes and functional capabilities, challenging the notion of neutrophils as a homogeneous population.^4^ Moreover, neutrophils exhibit impressive capacities to adapt their transcriptional programs and functional states in response to microenvironmental cues, termed neutrophil plasticity.^5^

This newfound appreciation of neutrophil heterogeneity and plasticity has profound implications for understanding their roles in health and disease. Beyond their canonical antimicrobial functions, neutrophils are now recognized as critical regulators of inflammation, immune responses, tissue repair, and tumor progression.^6^, ^7^ The ability of neutrophils to acquire distinct phenotypes and functions in response to local signals highlights their potential as therapeutic targets in a wide range of pathological conditions.^8^ However, harnessing the full potential of neutrophil‐targeted therapies requires a comprehensive understanding of the molecular mechanisms governing neutrophil heterogeneity and plasticity across different tissues and disease states.^9^, ^10^

This review explores the multidimensional landscape of neutrophil heterogeneity and plasticity, integrating insights from recent single‐cell transcriptomic studies, functional assays, and in vivo models. We discuss the critical dimensions of neutrophil heterogeneity, including maturation state, density, surface marker expression, and functional polarization, and highlight the microenvironmental drivers and molecular mechanisms underpinning neutrophil plasticity.^11^ Furthermore, we examine the role of neutrophil heterogeneity and plasticity in tissue and organ homeostasis, inflammation, infection, and cancer, emphasizing their potential as therapeutic targets.^12^, ^13^

The review is organized into six main sections, each focusing on a critical aspect of neutrophil biology. Section 2 delves into the multidimensional landscape of neutrophil heterogeneity, discussing the various axes along which neutrophil diversity manifests and the functional implications of distinct neutrophil subsets.^14^ Section 3 explores essential neutrophil functions’ signaling pathways, including recruitment, migration, antimicrobial activities, and cell fate decisions. In Section 4, we examine the emerging roles of neutrophils in tissue repair and regeneration, highlighting their contributions to wound healing, angiogenesis, stem cell regulation, and extracellular matrix (ECM) remodeling. Section 5 focuses on neutrophil plasticity in the context of tissue damage diseases, discussing the phenotypic and functional adaptations of neutrophils in acute injuries, chronic inflammatory conditions, and fibrotic disorders. In Section 6, we discuss the therapeutic targeting of neutrophils, presenting an overview of small molecule inhibitors, biologics, cell‐based therapies, and nanomedicine platforms aimed at modulating neutrophil functions in various disease contexts.

## 2 NEUTROPHIL HETEROGENEITY AND PLASTICITY

Neutrophils, the most abundant circulating leukocytes, have traditionally been viewed as a homogeneous population of short‐lived, terminally differentiated cells with a canonical role in antimicrobial defense. However, a paradigm shift has emerged over the past decade, revealing remarkable heterogeneity and plasticity within the neutrophil compartment. Advances in single‐cell technologies and functional assays have unveiled the existence of distinct neutrophil subsets with diverse phenotypes and functional capabilities, challenging the notion of neutrophils as a uniform population. Moreover, neutrophils exhibit impressive capacities to adapt their transcriptional programs and functional states in response to microenvironmental cues, termed neutrophil plasticity. This newfound appreciation of neutrophil heterogeneity and plasticity has profound implications for understanding their roles in health and disease.

### 2.1 Multidimensional landscape of neutrophil heterogeneity

Neutrophil heterogeneity manifests across multiple dimensions: maturation state, density, surface marker expression, and functional polarization. One well‐established axis of heterogeneity relates to the neutrophil maturation continuum. Contrary to the traditional view of neutrophils as terminally differentiated cells, they exhibit a spectrum of maturity ranging from immature progenitors to aged, senescent cells.^15^ Immature neutrophils, characterized by their banded nuclear morphology or low‐density properties, are mobilized from the bone marrow during emergency granulopoiesis and display enhanced inflammatory and oxidative burst capacities.^16^, ^17^ In contrast, aged neutrophils, marked by increased CXCR4 and decreased CD62L surface expression, exhibit impaired antimicrobial functions but a heightened propensity for neutrophil extracellular trap (NET) formation and immunomodulatory roles.^18^, ^19^

Neutrophils can be polarized into distinct functional states reminiscent of the M1/M2 paradigm in macrophages. Evidence supports the existence of proinflammatory “N1” neutrophils and anti‐inflammatory “N2” neutrophils, which differentially contribute to the initiation and resolution of inflammatory responses.^20^ N1 and N2 neutrophils can be distinguished immunophenotypically based on their differential expression of surface markers. N1 neutrophils, primed by inflammatory stimuli like LPS or IFN‐γ, exhibit increased expression of activation markers such as CD11b, CD66b, and CD64.^21^, ^22^ In contrast, N2 neutrophils, induced by anti‐inflammatory factors like transforming growth factor‐β (TGF‐β), interleukin‐4 (IL‐4), or glucocorticoids, display higher levels of CD16, CD163, and CD206, which are typically associated with an immunoregulatory phenotype.^23^, ^24^ Additionally, N1 neutrophils have been shown to express higher levels of the chemokine receptor CXCR2, which mediates their recruitment to sites of inflammation. On the other hand, N2 neutrophils exhibit increased expression of the chemokine receptor CXCR4, which is involved in their homing to the bone marrow and lymphoid organs, where they can exert immunomodulatory functions. This functional polarization enables neutrophils to orchestrate both the initiation and resolution phases of inflammatory responses.

Neutrophil heterogeneity also extends to their buoyancy, with low‐density neutrophils (LDNs) and normal‐density neutrophils (NDNs) exhibiting distinct phenotypic and functional properties. LDNs, enriched in inflammatory conditions, display an activated phenotype with enhanced production of reactive oxygen species (ROS), cytokines, and NETs, and increased expression of adhesion molecules and chemokine receptors.^25^, ^26^ In contrast, NDNs predominate in healthy individuals and exhibit more robust antimicrobial functions, including phagocytosis and degranulation.^24^, ^27^

Recent single‐cell transcriptomic analyses have further expanded the landscape of neutrophil heterogeneity, identifying distinct subsets based on their gene expression profiles.^4^, ^28^ These subsets encompass proinflammatory, anti‐inflammatory, and immunoregulatory populations with unique functional capabilities, such as differential cytokine production, phagocytic capacity, and interactions with other immune cells.^29^, ^30^ Moreover, neutrophils acquire tissue‐specific phenotypes and functions depending on the microenvironment they encounter.^31^ For instance, tumor‐associated neutrophils (TANs) exhibit distinct transcriptional signatures and functional properties that promote tumor progression, including producing proangiogenic factors, matrix‐remodeling enzymes, and immunosuppressive mediators.^32^, ^33^ Similarly, neutrophils in the lung microenvironment during acute respiratory distress syndrome (ARDS) display a proinflammatory phenotype characterized by increased production of cytokines, chemokines, and NETs, contributing to lung injury.^34^, ^35^

### 2.2 Microenvironmental drivers of neutrophil plasticity

The remarkable plasticity of neutrophils, enabling their transition between distinct phenotypic and functional states, is governed by a complex interplay of microenvironmental signals.^21^, ^36^ Key factors shaping neutrophil plasticity include inflammatory mediators, metabolic cues, and tissue‐specific signals.

Inflammatory mediators, such as cytokines, chemokines, and pathogen‐associated molecular patterns, DAMPs, are pivotal drivers of neutrophil polarization and functional reprogramming.^37^ Proinflammatory stimuli like LPS, tumor necrosis factor‐alpha (TNF‐α), and IFN‐γ promote the acquisition of an N1 phenotype characterized by enhanced antimicrobial functions and proinflammatory mediator production.^20^, ^21^ Conversely, anti‐inflammatory signals like TGF‐β, IL‐4, and glucocorticoids induce an N2 phenotype, favoring immunomodulatory and proresolving functions.^20^

Metabolic cues, including nutrient availability, oxygen tension, and metabolic intermediates, also profoundly influence neutrophil plasticity.^47^ Hypoxic microenvironments in inflamed or neoplastic tissues stabilize hypoxia‐inducible factor‐1alpha (HIF‐1α), driving a metabolic shift toward glycolysis and promoting proinflammatory neutrophil phenotypes.^38^, ^39^ In contrast, normoxic conditions favor oxidative phosphorylation and anti‐inflammatory neutrophil polarization.^40^ Metabolic intermediates like succinate, fumarate, and itaconate can modulate neutrophil functions by influencing epigenetic landscapes and signaling pathways.^41^, ^42^, ^43^

Tissue‐specific signals, including ECM components, stromal cell‐derived factors, and organ‐specific metabolites, further shape neutrophil plasticity.^44^ For instance, tumor‐derived factors like granulocyte‐colony‐stimulating factor (G‐CSF), vascular endothelial growth factor (VEGF), and hypoxia influence the phenotype and function of TANs.^20^, ^32^ The gut microbiome has also been implicated in modulating neutrophil phenotypes and functions by producing metabolites and modulating inflammatory pathways.^45^, ^46^

### 2.3 Molecular mechanisms underpinning neutrophil plasticity

The plasticity of neutrophils is underpinned by dynamic alterations in their transcriptional programs, mediated by a complex network of signaling pathways and epigenetic modifications. Metabolic reprogramming plays a central role in governing neutrophil plasticity, with proinflammatory stimuli inducing a shift toward aerobic glycolysis.^42^ At the same time, anti‐inflammatory cues promote oxidative phosphorylation and fatty acid oxidation.^41^ These metabolic adaptations are orchestrated by transcription factors like HIF‐1α and signaling pathways such as PI3K/Akt/mTOR^38^.

Signaling cascades involving mitogen‐activated protein kinases (MAPKs), nuclear factor‐kappa B (NF‐κB), and signal transducer and activator of transcription (STAT) proteins integrate environmental cues and drive transcriptional programs that shape neutrophil phenotypes and functions.^21^ For instance, the p38 MAPK pathway is crucial for inducing proinflammatory neutrophil responses,^36^ while the STAT3 pathway promotes anti‐inflammatory polarization.^20^

Epigenetic mechanisms, including histone modifications, DNA methylation, and noncoding RNAs, also regulate neutrophil plasticity by modulating the accessibility of gene regulatory regions and establishing distinct transcriptional programs.^47^ For example, the inhibition of histone deacetylases has promoted an anti‐inflammatory neutrophil phenotype.^37^ MicroRNAs (miRNAs), such as miR‐223, have been implicated in modulating neutrophil phenotypes and functions by targeting proinflammatory genes.^48^

Posttranslational modifications, including protein phosphorylation, acetylation, and ubiquitination, further fine‐tune the activity and stability of essential signaling proteins involved in neutrophil polarization and functional reprogramming.^43^ For instance, the ubiquitination and degradation of IκBα, an inhibitor of NF‐κB, is a critical step in activating proinflammatory neutrophil responses.^32^

Emerging evidence also suggests that neutrophils can undergo a form of innate immune memory, termed “trained immunity,” in response to certain stimuli.^44^ This phenomenon involves epigenetic reprogramming that enhances or suppresses neutrophil responses to subsequent challenges, potentially contributing to their functional plasticity.^40^

**FIGURE 1:**
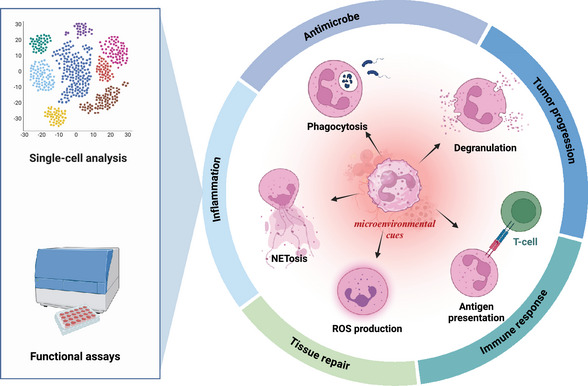
Neutrophils are activated and display a variety of functions in response to microenvironmental cues. Single‐cell technologies and functional assays unveil the existence of distinct neutrophil subsets with diverse phenotypes and functional capabilities. These neutrophils play key roles in the antimicrobe, inflammation, immune response, tissue repair, and tumor progression. Created with BioRender.com.

In conclusion, the past decade has witnessed a remarkable evolution in our understanding of neutrophil biology, revealing a complex landscape of heterogeneity and plasticity. Neutrophils comprise a spectrum of phenotypic and functional states dynamically shaped by diverse microenvironmental cues, metabolic adaptations, and epigenetic modifications. This multidimensional heterogeneity enables neutrophils to fulfill various roles beyond their canonical antimicrobial functions, including immunomodulation, tissue repair, and even the shaping of adaptive immune responses. The molecular mechanisms underpinning neutrophil plasticity involve a complex interplay of signaling pathways, transcriptional regulators, and epigenetic factors that fine‐tune neutrophil phenotypes and functions context‐dependently (Figure 1).

### 2.4 Developmental trajectory of neutrophil subsets

Neutrophils have traditionally been viewed as a homogeneous population of short‐lived, terminally differentiated cells with limited developmental plasticity. However, recent advances in single‐cell technologies and lineage tracing approaches have unveiled a complex landscape of neutrophil heterogeneity, with distinct subsets exhibiting diverse phenotypes, functions, and developmental trajectories.^49^ This section explores the current understanding of neutrophil development, focusing on the emergence of neutrophil subpopulations and the molecular mechanisms governing their differentiation and functional specialization.

#### 2.4.1 Granulopoiesis and the neutrophil differentiation cascade

Neutrophils originate from hematopoietic stem cells (HSCs) in the bone marrow through a tightly regulated process called granulopoiesis.^50^ This process involves a series of differentiation steps, starting with the commitment of HSCs to the myeloid lineage and progressing through various progenitor stages, including common myeloid progenitors, granulocyte‐monocyte progenitors (GMPs), and myeloblasts. The final stages of neutrophil differentiation involve the maturation of myeloblasts into promyelocytes, myelocytes, metamyelocytes, band cells, and finally, mature neutrophils.^51^, ^52^

The differentiation cascade is orchestrated by a complex network of transcription factors, cytokines, and growth factors that shape the transcriptional and epigenetic landscape of developing neutrophils. Key transcription factors driving neutrophil differentiation include C/EBPα, C/EBPε, and PU.1, which regulate the expression of genes involved in granule formation, cell cycle control, and effector functions.^53^, ^54^ Cytokines such as G‐CSF and granulocyte‐macrophage CSF (GM‐CSF) play crucial roles in promoting neutrophil survival, proliferation, and maturation.^55^, ^56^

Recent studies employing single‐cell RNA sequencing (scRNA‐seq) have provided unprecedented insights into the transcriptional dynamics of neutrophil differentiation. These analyses have revealed a continuum of differentiation states, with distinct transcriptional signatures associated with each stage of maturation.^57^, ^58^ Moreover, scRNA‐seq has uncovered previously unappreciated heterogeneity within the neutrophil compartment, identifying subpopulations with unique gene expression profiles and functional properties.^59^

#### 2.4.2 Emergence of neutrophil subsets during development

Accumulating evidence suggests that neutrophil heterogeneity is not merely a consequence of stochastic variation or activation states but rather reflects the existence of distinct developmental trajectories giving rise to specialized subpopulations. These subsets exhibit unique phenotypic and functional characteristics, suggesting that they may have evolved to fulfill specific roles in immune responses and tissue homeostasis.

One of the earliest examples of neutrophil heterogeneity was the identification of immature and mature neutrophil subsets based on their nuclear morphology and density properties.^60^ Immature neutrophils, characterized by their banded or ring‐shaped nuclei, are released from the bone marrow during stress or inflammation and exhibit enhanced inflammatory and oxidative burst capacities. In contrast, mature neutrophils, with their segmented nuclei, predominate in the circulation under homeostatic conditions and display more robust antimicrobial functions.

More recently, the advent of high‐dimensional flow cytometry and mass cytometry has enabled the identification of novel neutrophil subsets based on their surface marker expression profiles.^30^, ^61^ For instance, a distinct subset of CD177+ neutrophils has been identified in healthy individuals, comprising approximately 45–65% of the total neutrophil population. These CD177+ neutrophils exhibit enhanced phagocytic capacity and ROS production compared with their CD177− counterparts.^62^ Moreover, the proportion of CD177+ neutrophils has been shown to increase in various inflammatory conditions, such as bacterial infections and autoimmune disorders, suggesting that this subset may play a role in the pathogenesis of these diseases.^63^, ^64^

Another example of neutrophil heterogeneity is the identification of LDNs and NDNs based on their buoyancy properties.^65^ LDNs are enriched in inflammatory conditions and exhibit an activated phenotype with enhanced production of proinflammatory cytokines, NETs, and ROS. In contrast, NDNs predominate in healthy individuals and display more potent antimicrobial functions.^66^, ^67^ The developmental relationship between LDNs and NDNs remains a subject of ongoing investigation, with some studies suggesting that LDNs may represent a distinct lineage, while others propose that they arise from the activation or aging of NDNs.^68^

Recent studies employing scRNA‐seq and lineage tracing approaches have provided further insights into the developmental trajectories of neutrophil subsets. For example, a study by Wan et al. identified three distinct neutrophil subpopulations in the mouse bone marrow, termed “preneutrophils,” “immature neutrophils,” and “mature neutrophils,” each with unique transcriptional signatures and functional properties.^69^ Pseudotime analysis revealed a developmental progression from preneutrophils to mature neutrophils, with immature neutrophils representing an intermediate state.^70^ Interestingly, the authors also identified a subset of mature neutrophils that exhibited a proangiogenic phenotype, suggesting functional specialization within the mature neutrophil compartment.

Another study by Grieshaber‐Bouyer et al.^28^ used scRNA‐seq to characterize the neutrophil compartment across various tissues in mice. The authors identified a conserved neutrophil differentiation trajectory across tissues, with distinct subsets corresponding to different maturation stages. Moreover, they observed tissue‐specific adaptations in neutrophil gene expression profiles, suggesting that the local microenvironment can shape the functional specialization of neutrophils.

#### 2.4.3 Molecular mechanisms governing neutrophil subset differentiation

The molecular mechanisms underlying the emergence of neutrophil subsets during development are complex and multifaceted, involving the interplay of transcriptional regulators, epigenetic modifiers, and microenvironmental cues. Recent studies have begun to unravel the key players and pathways that drive the differentiation and functional specialization of neutrophil subpopulations.

One of the critical transcriptional regulators of neutrophil differentiation is the CCAAT/enhancer‐binding protein (C/EBP) family, particularly C/EBPα and C/EBPε.^71^ These transcription factors play essential roles in the commitment of myeloid progenitors to the granulocytic lineage and the subsequent maturation of neutrophils.^72^ Interestingly, the relative expression levels of C/EBPα and C/EBPε have been shown to influence the balance between immature and mature neutrophil subsets.^73^ High C/EBPα expression promotes the generation of immature neutrophils, while increased C/EBPε expression favors the production of mature neutrophils.

Another key transcriptional regulator of neutrophil development is growth factor independence 1 (Gfi1). Gfi1 is essential for the differentiation and survival of neutrophils, as evidenced by the severe neutropenia observed in Gfi1‐deficient mice.^74^ Recent studies have revealed that Gfi1 also plays a role in the functional specialization of neutrophil subsets. For example, a study by Ordoñez‐Rueda et al.^75^ demonstrated that Gfi1 regulates the expression of granule proteins and receptors in a subset‐specific manner. The authors found that Gfi1 deficiency led to the selective depletion of a mature neutrophil subset characterized by high expression of the Fc receptor FcγRIII and the granule protein Ngp.

Epigenetic mechanisms, such as DNA methylation and histone modifications, also contribute to the establishment and maintenance of neutrophil subset identities. For instance, a study by Ronnerblad et al.^76^ identified distinct DNA methylation patterns associated with different stages of neutrophil differentiation. The authors found that the promoter regions of genes involved in neutrophil effector functions, such as granule proteins and respiratory burst enzymes, underwent progressive demethylation during the course of differentiation. Moreover, they observed subset‐specific methylation signatures, with LDNs exhibiting a hypomethylated profile compared with NDNs, suggesting that epigenetic remodeling may underlie the functional specialization of these subsets.

The microenvironment in which neutrophils develop and reside also plays a crucial role in shaping their phenotypic and functional properties.^77^ Neutrophils are exposed to a wide array of soluble factors, cell–cell interactions, and physicochemical cues that can influence their differentiation and specialization. For example, the bone marrow niche provides a complex milieu of cytokines, growth factors, and stromal cell interactions that support neutrophil development and modulate their functional attributes.^78^ In particular, the balance between G‐CSF and GM‐CSF signaling has been shown to influence the generation of distinct neutrophil subsets, with G‐CSF favoring the production of mature, terminally differentiated neutrophils, while GM‐CSF promotes the expansion of immature, proinflammatory subsets.^79^

**FIGURE 2:**
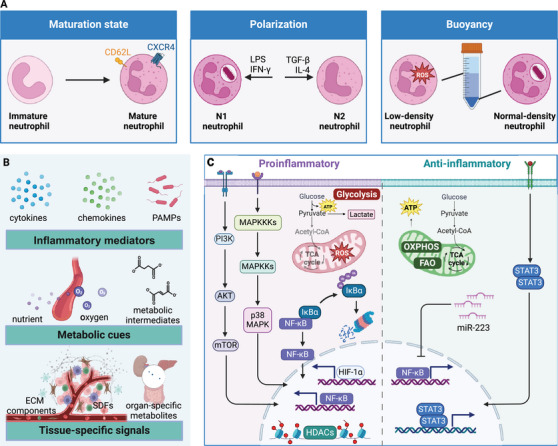
Neutrophil heterogeneity and plasticity. (A) Neutrophil heterogeneity manifests across multiple dimensions, including maturation state, functional polarization, density, and so on. (B) Key factors shaping neutrophil plasticity include inflammatory mediators, metabolic cues, and tissue‐specific signals. (C) The molecular mechanisms underpinning neutrophil plasticity involve a complex network of signaling pathways, transcriptional regulators, and epigenetic factors that fine‐tune neutrophil phenotypes and functions. Created with BioRender.com.

In conclusion, the unraveling of neutrophil heterogeneity and the delineation of distinct developmental trajectories have revolutionized our understanding of neutrophil biology and opened new avenues for therapeutic intervention. The identification of specialized neutrophil subsets with unique functional properties has revealed the complex and multifaceted roles of these cells in health and disease.^80^ As we continue to dissect the molecular mechanisms underlying neutrophil subset differentiation and specialization, we will be better positioned to develop targeted therapies that harness the full potential of these versatile immune cells. The journey ahead promises to yield exciting insights into the developmental origins of neutrophil heterogeneity and to transform our approach to the treatment of neutrophil‐mediated diseases (Figure 2).

## 3 SIGNALING PATHWAYS GOVERNING NEUTROPHIL FUNCTIONS

Neutrophils, the most abundant circulating leukocytes, serve as the first line of defense against invading pathogens and play a pivotal role in initiating and resolving inflammatory responses. Their remarkable functional versatility, encompassing phagocytosis, degranulation, NET formation, and cytokine secretion, is orchestrated by intricate signaling networks that precisely calibrate their activation, migration, antimicrobial activities, lifespan, and inflammatory outputs. Dysregulation of these signaling pathways can lead to impaired antimicrobial defense or excessive inflammation, contributing to the pathogenesis of various diseases. Over the past decade, significant advances have been made in elucidating the molecular mechanisms governing critical neutrophil functions, paving the way for the development of targeted therapies. This section provides an in‐depth review of the current understanding of signaling pathways regulating neutrophil recruitment, migration, antimicrobial functions, and cell fate, focusing on recent insights and their potential therapeutic implications.

### 3.1 Orchestration of neutrophil recruitment and migration

Efficient neutrophil recruitment to sites of infection or injury is a critical determinant of host defense and inflammatory responses. This complex process involves a tightly regulated cascade of events, including neutrophil release from bone marrow niches, margination within blood vessels, adhesion to activated endothelium, transendothelial migration, and chemotactic navigation through tissues.^36^, ^81^ Recent studies have shed light on the intricate signaling networks that govern each step of this migratory odyssey.

The mobilization of neutrophils from bone marrow reservoirs is triggered by inflammatory mediators, such as complement component C5a, IL‐8, and G‐CSF.^82^, ^83^ These stimuli activate neutrophil GPCRs, propagating signals through PLC, PI3K, and MAPK cascades.^84^ Downstream effectors, including the Rho GTPases Rac1/2 and Cdc42, orchestrate actin cytoskeletal remodeling, facilitating neutrophil detachment from bone marrow stromal cells.^85^, ^86^ Concurrently, G‐CSF‐mediated activation of the transcription factor STAT3 suppresses the expression of the retention chemokine CXCL12, further promoting neutrophil egress from the bone marrow.^83^, ^87^

As neutrophils enter the circulation, they undergo priming in response to inflammatory cues, enhancing their responsiveness to subsequent stimuli.^88^ Selectin‐mediated rolling along activated endothelium triggers signaling through Src family kinases (SFKs), Syk, and PLCγ2, leading to the upregulation of integrin activation states.^89^ Chemokine receptors, such as CXCR2, bind to immobilized IL‐8 gradients on the endothelial surface, propagating Gαi signals that amplify integrin affinity through the adaptor proteins talin‐1 and kindlin‐3.^90^, ^91^ Additionally, PI3Kγ generates phosphatidylinositol (3,4,5)‐trisphosphate (PIP3) lipids that stabilize integrin clustering and promote firm adhesion.^92^, ^93^

Neutrophil extravasation into tissues involves a complex interplay of adhesion receptors, chemokine sensing, and cytoskeletal remodeling. Engagement of endothelial intercellular adhesion molecule‐1 (ICAM‐1) triggers “outside‐in” signaling through the neutrophil β2 integrin Mac‐1, activating SFKs, Vav guanine nucleotide exchange factors (GEFs), and Rac/Cdc42 to coordinate actin polymerization.^85^, ^94^ Simultaneously, GPCR sensing of chemokine gradients, such as IL‐8 and leukotriene B4 (LTB4), activates PI3Kγ, generating PIP3 domains that direct cell polarity and protrusion through Rac, Cdc42, and WAVE regulatory complexes.^95^, ^96^ Rho/ROCK signaling mediates neutrophil tail detachment during transendothelial migration.^97^

Within tissues, neutrophils integrate multiple guidance cues, including chemokines, bacterial peptides, and ECM interactions, to navigate along chemotactic gradients. GPCRs activate Gαi proteins, such as Gαi2, which inhibit cAMP production, promoting cell polarity through Rac/Cdc42 and actin branching via WAVE complexes.^96^, ^98^ Gβγ subunits also activate PI3Kγ, generating PIP3 domains that recruit Rac GEFs and WAVE regulators to drive leading‐edge protrusion.^95^, ^99^ Simultaneously, receptor‐activated PLC generates IP3 to induce calcium fluxes that activate PKC isoforms and the Arp2/3 actin nucleator N‐WASP.^100^ Negative feedback loops involving phosphatases, such as PTEN, restrict protrusive activity to the leading edge, ensuring directional persistence.^101^

Recent studies have further elucidated the intricate signaling networks governing neutrophil migration. The atypical Rho GTPase RhoU has emerged as a critical regulator of neutrophil polarization and directional migration, acting through the Wiskott–Aldrich syndrome protein (WASP) Homology 2 And Myosin‐binding protein nucleation‐promoting factor to stimulate Arp2/3‐mediated actin polymerization at the leading edge.^102^ Additionally, the lipid chemoattractant S1P has been shown to induce neutrophil migration through S1P receptor signaling, activating Rac and Cdc42 GTPases and promoting integrin‐mediated adhesion.^103^

Integrating multiple guidance cues during neutrophil chemotaxis involves complex crosstalk between signaling pathways. For instance, the PI3K and MAPK pathways converge on the pseudokinase TRIB1, which acts as a molecular hub to coordinate neutrophil polarization and directional migration. Moreover, the Wnt/β‐catenin pathway has been implicated in regulating neutrophil trafficking, with Wnt ligands modulating the expression of adhesion molecules and chemokine receptors.^104^

Dysregulation of these signaling circuits can manifest in pathological contexts, such as impaired chemotaxis in chronic granulomatous disease or excessive neutrophil infiltration driving chronic inflammatory disorders.^21^, ^36^ Targeting key signaling nodes, such as PI3Kγ or Rho GTPases, represents a promising therapeutic strategy to modulate neutrophil migration in diverse disease settings^105^ (Table 1).

**TABLE 1:** Orchestration of neutrophil recruitment and migration.

The main stages of neutrophil recruitment and migration	Key mediators	Signaling pathways	Cytoskeletal remodeling	Regulatory mechanisms
Mobilization from bone marrow	‐ C5a, IL‐8, G‐CSF^82^, ^83^	‐ GPCR signaling via PLC, PI3K, MAPK	‐ Rac1/2, Cdc42‐mediated actin remodeling^85^, ^86^	‐ CXCL12 suppression by STAT3^83^, ^87^
Endothelial adhesion and rolling	‐ Selectins, integrins^89^, ^106^	‐ Src, Syk, PLCγ2 activation^89^, ^106^	‐ Integrin affinity modulation by talin‐1, kindlin‐3^90^, ^91^	‐ PI3K‐generated PIP3 stabilizes integrin clustering^92^, ^93^
Transendothelial migration	‐ ICAM‐1, Mac‐1^85^, ^94^	‐ SFKs, Vav GEFs, Rac/Cdc42^85^, ^94^	‐ Actin polymerization and protrusion^95^, ^96^	‐ Rho/ROCK‐mediated tail retraction
Chemotactic navigation	‐ Chemokines (IL‐8, LTB4)^95^, ^96^	‐ GPCR‐Gαi signaling, PI3Kγ, Rac/Cdc42^95,^ ^96^, ^98^	‐ Actin branching via WAVE complexes^96^, ^99^	‐ PTEN restricts protrusions to leading edge

Abbreviations: G‐CSF, granulocyte colony‐stimulating factor; GPCR, G protein‐coupled receptors; PLC, phospholipase C; MAPK, mitogen‐activated protein kinase; STAT, signal transducer and activator of transcription; ICAM, intercellular cell adhesion molecule; SFKs, Src family of protein tyrosine kinases; GEFs, GMP exchange factor; ROCK, Rho‐associated coiled‐coil‐containing protein kinase; WAVE, WASP‐family verprolin‐homologous proteins.

### 3.2 Signaling in antimicrobial functions

Neutrophils deploy a potent antimicrobial arsenal upon reaching sites of infection or tissue damage, including phagocytosis, degranulation, oxidative burst, and NET formation.^81^, ^107^ Various signaling pathways tightly regulate These diverse effector functions to ensure adequate pathogen clearance while minimizing collateral tissue damage.^84^

Phagocytosis, the engulfment of microbes or cellular debris, is initiated by ligating opsonic receptors, such as FcγRs or CRs.^108^ These receptors activate SFKs, including Hck, Fgr, and Lyn, which propagate signals through Syk and PLCγ2 to induce cytoskeletal rearrangements necessary for particle internalization.^85^, ^109^ Activated Rho GTPases, such as Rac1 and Cdc42, coordinate actin polymerization and membrane remodeling to form the phagocytic cup.^110^ Concurrently, PI3K generates PIP3 lipids that recruit and activate GEFs and effector proteins, further promoting actin assembly and phagosome formation.^111^

Following phagocytosis, neutrophils employ oxidative and nonoxidative mechanisms to eliminate internalized pathogens. The oxidative burst rapidly generates ROS by the NADPH oxidase complex, which assembles on the phagosomal membrane.^112^ The activation of the NADPH oxidase requires the phosphorylation and translocation of its cytosolic subunits, p47phox and p67phox, mediated by PKC and MAPK signaling.^113^ Rac2, a member of the Rac GTPase family, plays a crucial role in regulating NADPH oxidase assembly and activity.^114^

Degranulation, which releases preformed antimicrobial proteins and proteases from neutrophil granules, is another crucial effector mechanism.^115^ The mobilization of specific granule subsets is differentially regulated by calcium signaling and Rab GTPases.^116^, ^117^ Elevation of cytosolic calcium, triggered by PLC‐mediated IP3 generation or calcium influx through store‐operated channels, activates PKC and calcium‐dependent proteases like calpain, promoting granule fusion with the plasma membrane or phagosome.^118^, ^119^ Rab27a and Rab27b have been implicated in the differential exocytosis of neutrophil granule subsets, with Rab27a regulating azurophilic granules and Rab27b controlling specific and gelatinase granules.^120^, ^121^

NET formation, a recently discovered antimicrobial strategy, involves the extrusion of decondensed chromatin decorated with granule‐derived proteins to trap and kill microbes.^122^ The signaling pathways triggering NET formation, or NETosis, are complex and context‐dependent.^123^ In response to stimuli like phorbol esters or calcium ionophores, PKC activation and calcium influx induce the translocation of neutrophil elastase (NE) and myeloperoxidase (MPO) from granules to the nucleus, where they promote chromatin decondensation.^124^, ^125^ Rac2 and ROS generated by the NADPH oxidase are also critical for NETosis, with ROS‐mediated activation of the protein arginine deiminase 4 (PAD4) leading to histone citrullination and chromatin unfolding.^126^, ^127^ In contrast to the PKC‐ and ROS‐dependent pathway, NETosis induced by bacteria or immune complexes involves a distinct signaling cascade requiring integrin engagement, Src kinases, and Syk activation.^128^, ^129^

Recent studies have provided further insights into the molecular mechanisms regulating neutrophil antimicrobial functions. The cyclin‐dependent kinase (CDK) inhibitor roscovitine has been shown to enhance phagocytosis and bacterial killing by neutrophils, suggesting a novel role for CDKs in modulating phagocytic signaling.^130^ Additionally, the cGAS–STING pathway, traditionally associated with viral sensing, has been implicated in regulating NET formation, with STING activation promoting NETosis in response to cytosolic DNA.^131^

Negative regulatory mechanisms maintain the intricate balance between effective antimicrobial responses and excessive inflammation. For instance, the phosphatase SHIP‐1 breaks PI3K signaling, limiting phagocytosis and oxidative burst.^132^ The immunoreceptor tyrosine‐based inhibition motif (ITIM)‐containing receptors, such as SIRP‐α and PIR‐B, recruit phosphatases like SHP‐1 to attenuate ITAM‐mediated activation signals.^133^, ^134^ Dysregulation of these inhibitory pathways has been linked to neutrophil hyperactivation and tissue damage in various inflammatory disorders.^135^

Targeting signaling pathways involved in neutrophil antimicrobial functions offers therapeutic opportunities for infectious and inflammatory diseases. For example, pharmacological inhibition of PI3Kγ has shown promise in reducing neutrophil‐mediated tissue injury in sepsis and acute lung injury (ALI) models.^136^ Similarly, modulating the activity of Rho GTPases or their downstream effectors may provide a means to fine‐tune neutrophil responses in various disease contexts^137^, ^138^ (Table 2).

**TABLE 2:** Signaling in antimicrobial functions.

Antimicrobial arsenal	Key receptors/triggers	Signaling pathways	Cytoskeletal remodeling	Regulatory mechanisms
Phagocytosis	‐ FcγRs, CRs^108^, ^109^	‐ SFKs, Syk, PLCγ2^85^, ^109^	‐ Rac1, Cdc42‐mediated actin polymerization	‐ SHIP‐1 inhibits PI3K signaling^132^
		‐ PI3K generates PIP3		‐ ITIM‐containing receptors recruit SHP‐1^133^, ^134^
Oxidative Burst	‐ NADPH oxidase assembly^112^, ^113^	‐ PKC, MAPK activate p47phox, p67phox	‐ Rac2 regulates NADPH oxidase assembly	
Degranulation	‐ Calcium signaling, Rab GTPases^116^, ^117^	‐ PKC, calcium‐dependent proteases^118^, ^119^	‐ Rab27a regulates azurophilic granules	‐ Rab27b controls specific and gelatinase granules^121^
NET Formation	‐ Phorbol esters, calcium ionophores^124^, ^125^	‐ PKC, ROS‐mediated PAD4 activation^126^, ^127^	‐ Rac2 and ROS critical for NETosis^126^, ^127^	‐ cGAS–STING pathway regulates NET formation^131^
	‐ Bacteria, immune complexes^128^, ^129^	‐ Src kinases, Syk activation^128^, ^129^		

Abbreviations: SFKs, Src family of protein tyrosine kinases; SHIP‐1, SH2‐containing inositol phosphatase‐1; ITIM, immunoreceptor tyrosine‐based inhibition motif; MAPK, mitogen‐activated protein kinase; PKC, protein kinase C; NETs, neutrophil extracellular traps.

### 3.3 Signaling in neutrophil cell fate

In addition to their antimicrobial functions, neutrophils play a crucial role in shaping the inflammatory milieu and resolving inflammation. The lifespan and clearance of neutrophils are tightly regulated by signaling pathways that control apoptosis, survival, and efferocytosis.^139^ Moreover, recent studies have highlighted the active contribution of neutrophils to the resolution of inflammation through the production of specialized proresolving mediators (SPMs) and the adoption of distinct proresolving phenotypes.^140^

Neutrophil apoptosis is a tightly regulated process that ensures the timely removal of effete cells and prevents excessive inflammation. The intrinsic apoptotic pathway, triggered by intracellular stress or damage, is regulated by the balance between proapoptotic (e.g., Bax, Bak) and antiapoptotic (e.g., Mcl‐1, Bcl‐2) members of the Bcl‐2 family.^141^ Survival signals, such as GM‐CSF and IL‐8, promote neutrophil survival by activating PI3K/Akt and MAPK pathways, which upregulate the expression of antiapoptotic proteins.^142^, ^143^ In contrast, death receptors like Fas and TNF‐related apoptosis‐inducing ligand (TRAIL) receptors initiate the extrinsic apoptotic pathway, activating caspase‐8 and downstream effector caspases.^144^

The clearance of apoptotic neutrophils, or efferocytosis, is mediated by phagocytes like macrophages and is crucial for the resolution of inflammation.^145^ Apoptotic neutrophils release “find‐me” signals, such as ATP and lysophosphatidylcholine (LPC), which attract phagocytes through purinergic receptors and G2A, respectively.^146^, ^147^ The exposure of “eat‐me” signals, including phosphatidylserine (PS) and calreticulin, on the surface of apoptotic neutrophils, engages receptors like TIM‐4 and LRP1 on phagocytes, triggering Rac‐dependent actin rearrangements and subsequent engulfment.^148^, ^149^

Recent studies have underscored the active role of neutrophils in promoting inflammation resolution through the production of SPMs, such as lipoxins, resolvins, and protectins, which exert potent anti‐inflammatory and proresolving effects.^150^ The biosynthesis of SPMs is regulated by enzymes like 5‐lipoxygenase (5‐LOX) and 15‐LOX, whose expression and activity are modulated by cytokines and lipid mediators.^151^ SPMs act through specific G protein‐coupled receptors (GPCRs), such as ALX/FPR2 and GPR32, to inhibit neutrophil infiltration, enhance efferocytosis, and promote tissue repair.^152^, ^153^

Moreover, during the resolution phase of inflammation, neutrophils can transition from a proinflammatory to a proresolving phenotype, driven by local mediators such as annexin A1 and resolvin E1, which engage specific receptors and activate signaling pathways that reprogram neutrophil gene expression and function.^154^, ^155^ Proresolving neutrophils exhibit enhanced apoptosis, increased production of anti‐inflammatory cytokines like IL‐10, and the ability to promote tissue repair by releasing growth factors and proangiogenic mediators.

The manipulation of signaling pathways regulating neutrophil cell fate and phenotype represents an attractive therapeutic strategy for inflammatory diseases. For instance, promoting neutrophil apoptosis and efferocytosis through the modulation of Bcl‐2 family proteins or PS receptors could facilitate the resolution of chronic inflammation.^156^, ^157^ Additionally, harnessing the proresolving properties of SPMs and their receptors may offer a novel approach to treating inflammatory conditions characterized by impaired resolution, such as atherosclerosis and chronic obstructive pulmonary disease (COPD)^158^ (Table 3).

**TABLE 3:** Signaling in neutrophil cell fate decisions.

Cell fate decisions	Key signaling pathways	Molecular regulators	Functional consequences
Apoptosis regulation	‐ Intrinsic pathway triggered by intracellular stress/damage^141^	‐ Proapoptotic Bcl‐2 family members (Bax, Bak)^141^	‐ Timely removal of effete cells^139^
	‐ Extrinsic pathway initiated by death receptors (Fas, TRAIL)^144^	‐ Antiapoptotic Bcl‐2 family members (Mcl‐1, Bcl‐2)^141^	‐ Prevention of excessive inflammation^139^
Survival promotion	‐ GM‐CSF and IL‐8 activate PI3K/Akt and MAPK pathways^142^, ^143^	‐ Upregulation of antiapoptotic proteins^142^, ^143^	‐ Enhanced neutrophil lifespan and function^139^
Efferocytosis signaling	‐ “Find‐me” signals (ATP, LPC) attract phagocytes^146^, ^147^	‐ Purinergic receptors and G2A mediate chemotaxis^146^, ^147^	‐ Clearance of apoptotic neutrophils^145^
	‐ “Eat‐me” signals (PS, calreticulin) engage phagocyte receptors^148^, ^149^	‐ TIM‐4 and LRP1 receptors trigger engulfment^148^, ^149^	‐ Resolution of inflammation^145^
Proresolving phenotype induction	‐ SPMs (lipoxins, resolvins, protectins) act via GPCRs^150^, ^152^, ^153^	‐ ALX/FPR2 and GPR32 receptors mediate SPM effects^152^, ^153^	‐ Enhanced efferocytosis and tissue repair^150^
	‐ Annexin A1 and resolvin E1 reprogram neutrophil gene expression^154^, ^155^	‐ Increased production of IL‐10 and other anti‐inflammatory mediators	‐ Transition to anti‐inflammatory state^154^, ^155^

Abbreviations: GM‐CSF, granulocyte‐macrophage colony‐stimulating factor; PS, phosphatidylserine; TIM‐4, T cell immunoglobulin domain and mucin domain‐4; LRP1, LDL receptor related protein 1.

**FIGURE 3:**
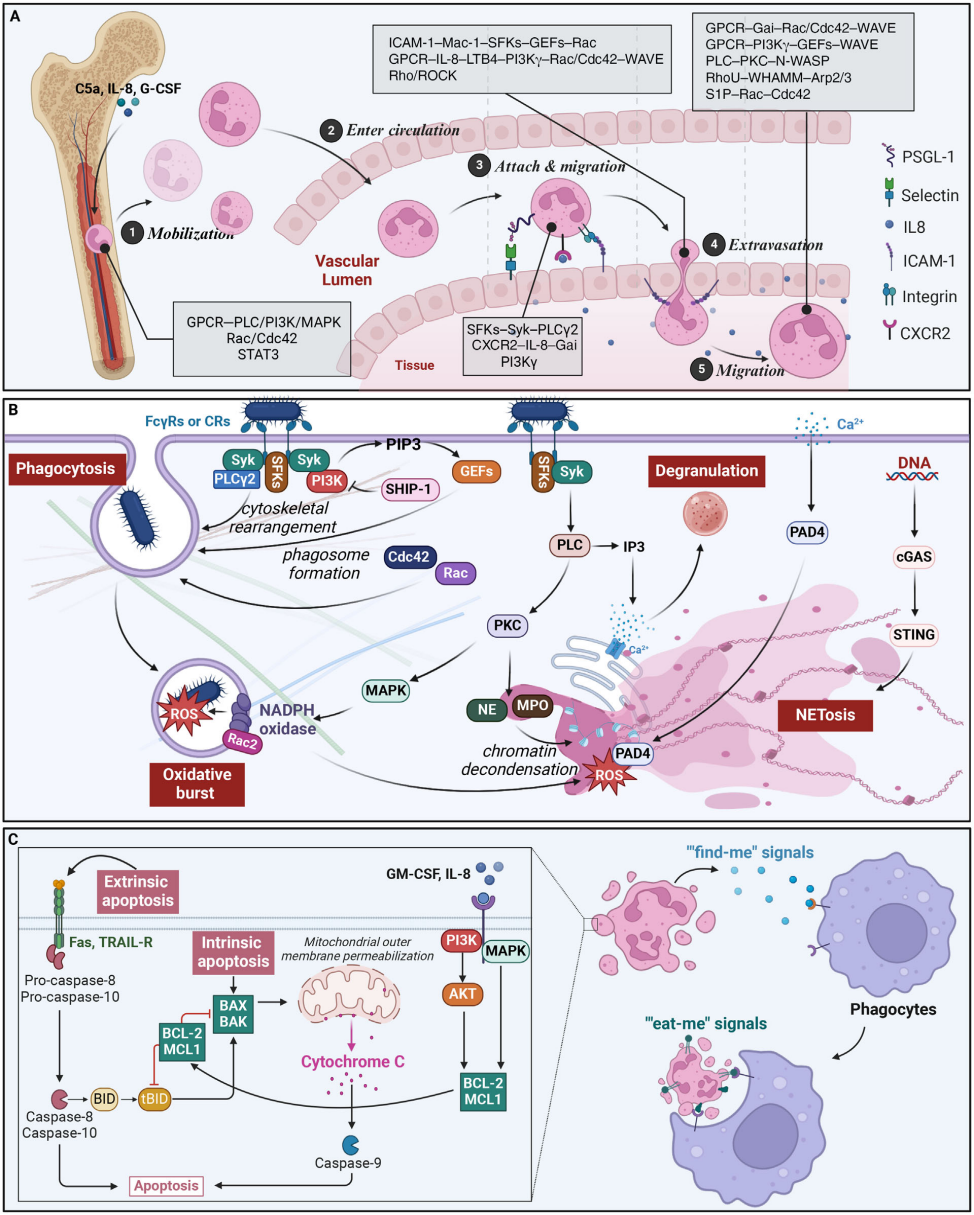
Signaling pathways governing neutrophil functions. (A) Neutrophil is recruited to infection or injury sites via a series of events, including mobilization from bone marrow niches, enter into blood vessels, adhesion to endothelium, migration, and extravasation into tissues. (B) Upon reaching infection or injury sites, neutrophils deploy a potent antimicrobial arsenal, including phagocytosis, degranulation, oxidative burst, and NETosis. (C) Neutrophil lifespan and clearance are tightly regulated by signaling pathways controlling apoptosis, survival, and efferocytosis. Created with BioRender.com.

In conclusion, the past decade has witnessed significant advances in understanding the signaling pathways governing neutrophil functions, from their recruitment and migration to their antimicrobial activities and cell fate decisions. These signaling networks exhibit remarkable complexity and plasticity, enabling neutrophils to mount tailored responses to diverse inflammatory challenges while maintaining tissue homeostasis. Dysregulation of these pathways underlies the pathogenesis of various infectious, inflammatory, and autoimmune diseases, highlighting the therapeutic potential of targeting neutrophil signaling. As our knowledge of the molecular mechanisms controlling neutrophil functions continues to expand, it opens up new avenues for developing precision therapies that harness the multifaceted roles of these essential immune cells in health and disease (Figure 3).

## 4 ROLE OF NEUTROPHIL PLASTICITY IN THE MAINTENANCE OF NORMAL PHYSIOLOGICAL HOMEOSTASIS

Neutrophils, the most abundant white blood cells in circulation, have traditionally been viewed as short‐lived, terminally differentiated cells with limited plasticity. However, recent research has unveiled neutrophils' remarkable adaptability and functional diversity, challenging this long‐held paradigm.

Neutrophils exhibit a spectrum of phenotypes and functions that extend far beyond their canonical role in innate immunity and inflammation. They can fine‐tune their responses based on microenvironmental cues, engaging in complex crosstalk with other immune cells, epithelial cells, and the microbiota. This plasticity allows neutrophils to mount tailored responses against pathogens while preserving tissue integrity and promoting the resolution of inflammation.

Moreover, neutrophils play a crucial role in shaping the composition and diversity of the microbiome at various barrier sites, such as the gut, skin, and lungs.^159^, ^160^ Through selective antimicrobial responses and the release of immunomodulatory factors, neutrophils actively maintain a delicate balance between the host and its resident microbes.^161^ Beyond their roles in immune and microbial homeostasis, neutrophils have emerged as critical regulators of metabolic health. They exhibit remarkable adaptability in response to metabolic cues, modulating their phenotypes and functions to regulate adipose tissue homeostasis, glucose metabolism, lipid metabolism, and liver function. The circadian regulation of neutrophil activity adds another layer of complexity to their metabolic roles.

### 4.1 Neutrophil plasticity maintaining immune homeostasis

Neutrophils are the most abundant leukocytes in the human blood and play a crucial role in innate immunity. Traditionally, neutrophils have been considered short‐lived effector cells that rapidly migrate to sites of inflammation, where they perform their primary functions, such as phagocytosis, degranulation, and release of NETs.^36^ However, recent studies have revealed that neutrophils exhibit remarkable plasticity and heterogeneity, adapting their phenotype and function in response to various microenvironmental cues.^15^, ^47^ Single‐cell transcriptome profiling has further highlighted the heterogeneity of neutrophils in homeostasis and infection, revealing distinct subpopulations with specialized functions.^4^ Moreover, tissue environments can shape neutrophil fates and functions, leading to the co‐option of neutrophils for tissue‐specific roles.^31^

#### 4.1.1 Dynamic regulation of neutrophil lifespan and turnover

Neutrophil homeostasis is maintained through a tightly regulated balance between granulopoiesis, release from the bone marrow, trafficking, and clearance.^162^ In vivo labeling with 2H2O has revealed that human neutrophils have a circulating half‐life of only 5.4 days.^163^ To maintain neutrophil numbers, the bone marrow produces approximately 5–10 × 10^10^ neutrophils daily in a healthy adult.^36^ Circulating neutrophils undergo spontaneous apoptosis, a process essential for maintaining immune homeostasis.^164^ Apoptotic neutrophils are cleared by phagocytes, such as macrophages and dendritic cells, through efferocytosis.^165^ Apoptotic neutrophils release “find‐me” and “eat‐me” signals that promote their recognition and uptake by phagocytes.^166^ Efferocytosis triggers the release of anti‐inflammatory mediators, such as TGF‐β, prostaglandin E2 (PGE2), and platelet‐activating factor (PAF), contributing to the resolution of inflammation.^167^, ^168^ When the macrophage clearance system is overwhelmed, neutrophils can sometimes engage in cannibalism. In this process, viable neutrophils phagocytose apoptotic neutrophils as a backup mechanism to prevent the release of toxic contents from necrotic cells.^169^ Impaired efferocytosis has been implicated in the pathogenesis of various chronic inflammatory disorders, such as COPD, asthma, and cystic fibrosis.^170^ The lifespan of neutrophils can be extended by proinflammatory mediators, such as GM‐CSF, IL‐1β, and LPS.^171^ GM‐CSF delays neutrophil apoptosis through the activation of the PI3K and ERK pathways.^143^ In contrast, proresolving lipid mediators, such as resolvins and protectins, promote neutrophil apoptosis and enhance efferocytosis, facilitating the resolution of inflammation.^150^, ^172^

#### 4.1.2 Neutrophil plasticity in immune surveillance

Recent studies have revealed that neutrophils are not only recruited to sites of inflammation but also reside in various tissues under steady‐state conditions, contributing to immune surveillance and tissue homeostasis.^173^ These tissue‐resident neutrophils exhibit unique phenotypes and functions compared with circulating neutrophils. For instance, lung‐resident neutrophils display a distinct crawling behavior and contribute to host defense against pathogens.^174^, ^175^ Neutrophils can also rapidly adapt their phenotype and function in response to microenvironmental cues, such as microbial products, cytokines, and tissue‐derived factors.^21^ For example, neutrophils exposed to low concentrations of LPS or other microbial products undergo transcriptional changes that enhance their survival, priming them for an enhanced response to subsequent stimuli.^176^ Neutrophils can also acquire an anti‐inflammatory or proresolving phenotype in response to apoptotic cells or anti‐inflammatory mediators. Phagocytosis of apoptotic neutrophils by macrophages induces a proresolving phenotype characterized by increased expression of scavenger receptors and production of TGF‐β.^177^ Similarly, exposure to anti‐inflammatory mediators, such as annexin A1, promotes a proresolving neutrophil phenotype with increased output of SPMs.^178^

#### 4.1.3 Neutrophil‐mediated regulation of other immune cells

In addition to their direct antimicrobial functions, neutrophils orchestrate immune responses by interacting with and modulating the function of other immune cells, including macrophages, dendritic cells, and lymphocytes.^21^ Neutrophils release chemokines and cytokines that attract and activate monocytes and macrophages, such as CCL2, CCL3, and CCL5.^179^ Neutrophil‐derived granule proteins, such as azurocidin and LL‐37, promote monocyte and dendritic cell recruitment and maturation.^180^, ^181^ Neutrophils can also directly trans‐differentiate into a distinct population of antigen‐presenting cells called “neutrophil‐DC hybrids” that can prime T cell responses.^182^ Apoptotic neutrophils and their phagocytosis by macrophages are crucial in shaping macrophage phenotype and function. Ingestion of apoptotic neutrophils by macrophages induces an anti‐inflammatory and proresolving phenotype, characterized by the release of TGF‐β, IL‐10, and SPMs.^150^, ^168^ This process is essential for the resolution of inflammation and the restoration of tissue homeostasis. In contrast, impaired efferocytosis can lead to neutrophil necrosis and the release of DAMPs, which promote macrophage activation and perpetuate inflammation.^183^ Neutrophils also modulate adaptive immunity by interacting with T and B lymphocytes. Activated neutrophils express major histocompatibility complex class II molecules and costimulatory receptors, enabling them to present antigens and stimulate T cell proliferation and differentiation.^184^ Neutrophils can also release cytokines, such as IL‐12, that promote Th1 differentiation or IL‐4 that supports Th2 responses.^185^, ^186^ In addition, neutrophils can stimulate B cell survival, proliferation, and antibody production by releasing B cell‐activating factor and a proliferation‐inducing ligand.^187^, ^188^

### 4.2 Neutrophil plasticity in the maintenance of microbiome homeostasis

Neutrophils are the most abundant circulating leukocytes in humans and play a crucial role in innate immunity. As the first line of defense against invading pathogens, neutrophils employ a diverse arsenal of antimicrobial strategies, including phagocytosis, degranulation, and the release of NETs.^122^ While traditionally viewed as short‐lived effector cells with a primary function in pathogen elimination, recent evidence suggests neutrophils also play a critical role in shaping and maintaining the host microbiome.

#### 4.2.1 Neutrophils as regulators of the microbiome

The gut microbiome is a complex ecosystem that plays a vital role in human health, and neutrophils have emerged as crucial regulators of this community. Neutrophil depletion in mice has been shown to lead to alterations in the gut microbiota, with an expansion of potentially pathogenic bacteria and a reduction in beneficial commensal species.^159^ Neutrophils in the gut exhibit a remarkable ability to distinguish between commensal and pathogenic strains of bacteria, selectively eliminating the latter while preserving the former. This selective targeting is mediated, in part, by the production of antimicrobial peptides and ROS that preferentially target pathogenic bacteria.^161^ Furthermore, neutrophil‐derived α‐defensins have been shown to modulate the balance between the two dominant phyla in the gut, Bacteroides and Firmicutes, by selectively inhibiting the growth of certain Bacteroides species while promoting the expansion of Firmicutes.^189^ The skin is another site where neutrophils play a critical role in microbiome homeostasis. Neutrophils in the skin have been shown to regulate the colonization and growth of commensal bacteria, such as Staphylococcus epidermidis.^160^ In the context of infection, neutrophils mount targeted responses against pathogens like Staphylococcus aureus while maintaining tolerance to commensal species.^190^ The crosstalk between neutrophils and T cells has also been identified as a key factor in maintaining a healthy skin microbiome, with disruption of this interaction leading to dysbiosis and increased susceptibility to infections.^160^ Although less well characterized than gut and skin, the lung microbiome is increasingly recognized as an essential determinant of respiratory health. Neutrophils in the lungs of healthy mice exhibit a distinct transcriptional profile compared with those in circulation, suggesting a specialized role in microbiome homeostasis.^191^ Neutrophils in the lung have been shown to modulate their degranulation and NET formation in response to specific bacterial stimuli, such as Pseudomonas aeruginosa and Streptococcus pneumoniae.^192^ Moreover, neutrophils in the lung produce lipoxin A4, a SPM that promotes epithelial repair and regeneration following injury, highlighting their role in maintaining tissue homeostasis.^193^

#### 4.2.2 Mechanisms of neutrophil–microbiome interactions

Neutrophils employ a diverse array of antimicrobial effector functions to shape the microbiome. These include phagocytosis, degranulation, and the release of NETs.^122^ Phagocytosis allows neutrophils to engulf and eliminate pathogenic bacteria, while degranulation releases antimicrobial peptides and enzymes that target specific microbial structures. NETs, composed of decondensed chromatin and antimicrobial proteins, can trap and kill extracellular bacteria. Importantly, neutrophils in different anatomical sites exhibit distinct patterns of antimicrobial effector functions tailored to the specific challenges posed by the local microbiome.^161^, ^192^ In addition to their direct antimicrobial activities, neutrophils shape the microbiome through immunomodulatory functions. Neutrophil‐derived cytokines, such as IL‐1β and IL‐22, play a critical role in host defense against Pseudomonas aeruginosa infection and promote the growth of beneficial commensal bacteria, such as Lactobacillus and Bifidobacterium, while suppressing the expansion of pathogenic species.^194^ Neutrophils also use crosstalk with other immune cells, such as T cells and innate lymphoid cells, to maintain microbiome homeostasis.^160^ Moreover, neutrophil‐derived proteases, such as NE, can modulate the expression and localization of tight junction proteins, influencing epithelial permeability and thus the access of microbial products to the host immune system.^195^ The microbiome produces a wide range of metabolites that can influence neutrophil function. Short‐chain fatty acids (SCFAs), particularly butyrate, have suppressed neutrophil migration and ROS production, promoting a more tolerogenic phenotype.^196^ Similarly, indole‐3‐aldehyde, a tryptophan metabolite produced by the gut microbiota, attenuates neutrophil infiltration and tissue damage in a mouse model of colitis.^197^ These findings highlight the complex bidirectional relationship between neutrophils and the microbiome, with microbial metabolites shaping neutrophil function and neutrophils shaping the microbiome's composition and activity.

#### 4.2.3 Neutrophil plasticity and microbiome homeostasis

One of the most remarkable features of neutrophils is their plasticity, which allows them to adopt distinct functional states in response to different microenvironmental cues. Exposure to microbial products, such as LPS and peptidoglycan, can induce a state of tolerance or priming in neutrophils, modulating their subsequent responses to infectious challenges.^198^ This plasticity enables neutrophils to mount appropriate responses to commensal and pathogenic microbes, maintaining a delicate balance between tolerance and inflammation. Neutrophils also contribute to microbiome homeostasis by promoting the integrity of epithelial barriers. Neutrophil‐derived IL‐22 stimulates the production of antimicrobial peptides and mucins by epithelial cells, reinforcing the mucosal barrier in the gut.^199^ Moreover, neutrophils promote the proliferation and survival of intestinal epithelial cells, enhancing barrier integrity. These barrier‐protective functions of neutrophils are essential for maintaining a healthy host‐microbiome interface, preventing the translocation of pathogenic microbes and their products across epithelial surfaces.

### 4.3 Neutrophil plasticity in the regulation of healthy metabolic homeostasis

Neutrophils play a crucial role in maintaining metabolic homeostasis, as demonstrated by their involvement in regulating various aspects of metabolism.^200^, ^201^ These cells exhibit remarkable plasticity in response to metabolic cues, adapting their phenotypes and functions to meet the specific needs of the tissue microenvironment.^202^, ^203^ Furthermore, neutrophil activity and metabolism are subject to circadian regulation, adding another layer of complexity to their role in metabolic homeostasis.^204^, ^205^

#### 4.3.1 Neutrophils in adipose tissue homeostasis

Neutrophils are essential regulators of adipose tissue health and function.^202^, ^206^ In lean adipose tissue, neutrophils exhibit an anti‐inflammatory phenotype, characterized by the production of IL‐10 and other anti‐inflammatory cytokines, which contribute to maintaining insulin sensitivity and adipocyte function.^207^, ^208^ However, in obesity, neutrophils infiltrate the adipose tissue and acquire a proinflammatory phenotype, secreting cytokines such as TNF‐α and IL‐6, which promote insulin resistance and adipose tissue dysfunction.^209^, ^210^ Various factors, including adipokines, fatty acids, and hypoxia, regulate neutrophils’ plasticity in adipose tissue. For example, adiponectin, an anti‐inflammatory adipokine, has been shown to promote an anti‐inflammatory neutrophil phenotype, enhancing IL‐10 production and reducing proinflammatory cytokine secretion.^211^ In contrast, saturated fatty acids can activate neutrophils through TLR4 signaling, inducing a proinflammatory phenotype.^212^ Furthermore, hypoxia, a common feature of obese adipose tissue, can modulate neutrophil function by stabilizing HIF‐1α, leading to enhanced neutrophil survival and proinflammatory cytokine production.^213^

#### 4.3.2 Neutrophil plasticity in glucose metabolism

Neutrophils are also sensitive to changes in glucose metabolism, and their phenotypes and functions are altered in hyperglycemic conditions. In diabetes, neutrophils undergo functional changes, including enhanced ROS production, increased adhesion to endothelial cells, and delayed apoptosis.^214^, ^215^ These alterations contribute to the development of insulin resistance and the progression of diabetes‐related complications.^216^, ^217^ The plasticity of neutrophils in glucose metabolism is regulated by various signaling pathways, including insulin signaling and MAPK/ERK activation. Insulin can modulate neutrophil function by activating the PI3K/Akt pathway, leading to increased glucose uptake and enhanced neutrophil survival.^218^ In contrast, hyperglycemia can activate the MAPK/ERK pathway in neutrophils, promoting ROS production and proinflammatory cytokine secretion.^219^ Furthermore, neutrophil‐derived factors, such as NE, can impair insulin signaling in hepatocytes and adipocytes, contributing to insulin resistance.^203^ NETs are also implicated in the pathogenesis of type 2 diabetes, as they promote inflammation and impair glucose tolerance.^220^

#### 4.3.3 Lipid metabolism and neutrophil function

Lipid metabolism is another vital aspect of neutrophil plasticity in metabolic homeostasis. Neutrophils express a wide array of lipid receptors, including receptors for prostaglandins, leukotrienes, and lipoxins, allowing them to respond to various lipid signals and adapt their phenotypes accordingly.^221^, ^222^ For example, PGE2 can modulate neutrophil function by activating EP2 and EP4 receptors, leading to increased cAMP levels and suppression of neutrophil activation.^223^ In contrast, LTB4 can promote neutrophil chemotaxis, adhesion, and ROS production by activating BLT1 and BLT2 receptors.^224^ The balance between proinflammatory lipid mediators and anti‐inflammatory SPMs is crucial for maintaining neutrophil homeostasis and resolving inflammation.^150^ Neutrophils also actively participate in lipid metabolism by secreting lipid‐binding proteins, such as lipocalin‐2 (LCN2). LCN2 has been shown to promote thermogenesis and energy expenditure in brown adipose tissue by activating the β3‐adrenergic receptor pathway.^225^

#### 4.3.4 Neutrophils in liver metabolic homeostasis

Neutrophils play a crucial role in maintaining liver metabolic homeostasis. In healthy liver tissue, neutrophils exhibit a quiescent phenotype, characterized by low expression of activation markers and limited production of proinflammatory cytokines.^226^ However, neutrophils can acquire a proinflammatory phenotype in the context of liver injury or metabolic stress, contributing to the development of liver inflammation and insulin resistance.^202^, ^227^ Various cytokines and signaling pathways regulate the plasticity of neutrophils in the liver. IL‐17, for example, can activate neutrophils and promote their recruitment to the liver, contributing to the pathogenesis of nonalcoholic steatohepatitis (NASH).^227^ In contrast, IL‐22 can protect against liver injury by inducing the expression of antiapoptotic and antioxidant genes in hepatocytes.^228^ Neutrophil‐derived factors, such as MPO, can also modulate liver metabolic homeostasis. MPO has been shown to impair insulin signaling in hepatocytes, contributing to the development of insulin resistance.^203^ Furthermore, neutrophils can activate Kupffer cells by releasing cytokines and DAMPs, promoting liver inflammation and metabolic dysfunction.^229^

#### 4.3.5 Circadian regulation of neutrophil function and metabolism

Neutrophils exhibit diurnal variations in their numbers, phenotypes, and activities, regulated by the circadian clock machinery.^205^, ^230^ In humans, neutrophil counts peak during the day and reach their lowest levels at night, coinciding with the rest‐activity cycle.^231^ The circadian regulation of neutrophil function is mediated by various clock genes, such as BMAL1, CLOCK, and REV‐ERBα, which control the expression of key genes involved in neutrophil trafficking, activation, and effector functions.^232^ Disruption of the circadian clock, either by genetic manipulation or environmental factors (e.g., shift work, jet lag), can lead to altered neutrophil phenotypes and impaired immune function, contributing to the development of metabolic disorders.^232^, ^233^ Neutrophils also participate in the regulation of circadian rhythms in metabolic tissues. For example, neutrophil‐derived factors can modulate the expression of clock genes in hepatocytes, contributing to the synchronization of liver metabolic rhythms.^205^ Furthermore, neutrophil infiltration into adipose tissue exhibits circadian oscillations, contributing to the diurnal regulation of adipose tissue inflammation and insulin sensitivity.

In summary, neutrophil plasticity is a rapidly evolving concept that highlights these innate immune cells’ remarkable adaptability and functional diversity. From being static, short‐lived entities, neutrophils exhibit a spectrum of phenotypes and functions dynamically regulated by microenvironmental cues. This plasticity allows neutrophils to fine‐tune their responses in the context of immune regulation, microbiome balance, and metabolic homeostasis. In the realm of immunity, neutrophils engage in complex crosstalk with other immune cells, mounting tailored responses against pathogens while promoting the resolution of inflammation. They also actively shape the composition and diversity of the microbiome at various barrier sites through selective antimicrobial responses and immunomodulatory factor release. Moreover, neutrophils have emerged as critical regulators of metabolic health, adapting their phenotypes to modulate adipose tissue homeostasis, glucose and lipid metabolism, and liver function. As the field of neutrophil plasticity continues to expand, it opens new therapeutic avenues for diseases characterized by immune dysregulation, microbial imbalance, and metabolic dysfunction. Harnessing neutrophil adaptability may be key to developing targeted interventions that promote homeostasis and prevent pathological conditions across various physiological systems.

## 5 NEUTROPHIL PLASTICITY IN TISSUE DAMAGE DISEASES

Neutrophils, the most abundant circulating leukocytes, are the first responders to tissue injury and play a pivotal role in initiating, propagating, and resolving inflammatory responses. While their primary function is to eliminate invading pathogens and clear cellular debris, the multifaceted roles of neutrophils in tissue damage diseases have come to light in recent years. Neutrophil plasticity, the ability to adapt and acquire distinct phenotypes in response to microenvironmental cues, has emerged as a critical determinant of their contribution to the pathogenesis of various acute injuries, chronic injuries, and fibrotic conditions. This section explores the role of neutrophil plasticity across a spectrum of tissue damage diseases, highlighting this phenomenon's molecular mechanisms, functional consequences, and therapeutic implications.

### 5.1 Neutrophil plasticity in acute tissue injuries

Acute tissue injuries, such as those resulting from ischemia–reperfusion, trauma, or chemical insults, trigger a rapid and robust inflammatory response characterized by the massive infiltration of neutrophils.^36^ While neutrophils play a crucial role in the initial stages of tissue repair by clearing cellular debris and releasing growth factors, their excessive activation and prolonged presence can exacerbate tissue damage and impair the resolution of inflammation.^140^

In the context of ALI and ARDS, neutrophils have been shown to exhibit remarkable plasticity, adopting distinct phenotypes that contribute to the pathogenesis of these conditions.^234^ Upon exposure to proinflammatory stimuli, such as LPS or cytokines like IL‐8 and TNF‐α, neutrophils acquire a hyper‐activated phenotype characterized by enhanced production of ROS, proteases, and proinflammatory mediators.^235^ These activated neutrophils cause direct tissue damage by releasing cytotoxic molecules and exacerbate inflammation by recruiting additional immune cells to the injury site.^236^

Moreover, neutrophils in ALI/ARDS exhibit an increased propensity for NET formation, known as NETosis.^237^ NETs, composed of decondensed chromatin decorated with antimicrobial proteins, can trap and kill pathogens and contribute to lung injury by promoting alveolar–capillary barrier dysfunction, thrombosis, and fibrosis.^123^ The molecular mechanisms driving NET formation in ALI/ARDS involve the activation of key signaling pathways, such as the Raf–MEK–ERK cascade and the PI3K–Akt axis, which induce the translocation of NE and MPO to the nucleus, facilitating chromatin decondensation.^238^

In contrast to the detrimental effects of hyper‐activated neutrophils, emerging evidence suggests that neutrophils can acquire an anti‐inflammatory and proresolving phenotype during the resolution phase of ALI/ARDS.^239^ These neutrophils, often referred to as “N2” or “polymorphonuclear myeloid‐derived suppressor cells,” exhibit increased expression of immunomodulatory molecules like arginase‐1, TGF‐β, and IL‐10.^240^ N2 neutrophils suppress T cell proliferation, promote the efferocytosis of apoptotic cells, and facilitate tissue repair, thereby contributing to the resolution of inflammation and the restoration of lung homeostasis.^241^

The plasticity of neutrophils has also been implicated in the pathogenesis of AKI, a common complication of sepsis, ischemia–reperfusion, and nephrotoxic insults.^242^ In AKI, neutrophils infiltrate the kidney and acquire a proinflammatory phenotype, releasing ROS, proteases, and cytokines that damage renal tubular cells and promote microvascular dysfunction.^243^ Additionally, neutrophils in AKI exhibit enhanced NET formation, which can obstruct renal tubules, promote thrombosis, and exacerbate inflammation.^244^

Recent studies have highlighted the molecular mechanisms driving neutrophil plasticity in AKI. The activation of TLRs, mainly TLR4, by DAMPs released from injured renal cells has been shown to induce a proinflammatory neutrophil phenotype.^245^ Furthermore, the chemokine receptor CXCR2 and its ligands, such as CXCL1 and CXCL2, play a crucial role in neutrophil recruitment and activation in AKI.^246^ Targeting these signaling pathways may offer therapeutic opportunities to modulate neutrophil plasticity and attenuate renal injury.^247^

In the context of ischemic stroke, neutrophils have been shown to exhibit distinct phenotypes that contribute to both brain injury and repair.^248^ In the acute phase of stroke, neutrophils acquire a proinflammatory phenotype characterized by releasing ROS, proteases, and proinflammatory cytokines, which exacerbate blood–brain barrier disruption, neuronal death, and cerebral edema.^249^ Moreover, neutrophils in ischemic stroke display an increased propensity for NET formation, which can promote thrombosis, microvascular occlusion, and neuroinflammation.^250^

However, recent evidence suggests neutrophils can also acquire a neuroprotective phenotype in the subacute and chronic phases of stroke.^251^ These neutrophils exhibit increased expression of anti‐inflammatory mediators, such as IL‐10 and TGF‐β, and promote the clearance of cellular debris, thereby facilitating tissue repair and functional recovery.^252^ The molecular mechanisms underlying this phenotypic switch involve the activation of the transcription factor Nrf2, which induces the expression of antioxidant and anti‐inflammatory genes.^253^

### 5.2 Neutrophil plasticity in chronic tissue injuries

Chronic tissue injuries, such as those associated with nonhealing wounds, COPD, and inflammatory bowel disease (IBD), are characterized by persistent inflammation and impaired tissue repair. Neutrophil plasticity has emerged as a key factor in the pathogenesis of these conditions, with distinct neutrophil phenotypes contributing to the perpetuation of inflammation and the failure of resolution.^14^, ^254^ The role of neutrophils in chronic tissue injuries has been extensively studied, highlighting their potential as therapeutic targets.

In the context of nonhealing wounds, such as diabetic foot ulcers and pressure ulcers, neutrophils exhibit a dysfunctional phenotype characterized by impaired phagocytosis, reduced ROS production, and decreased chemotactic ability.^255^ These functional deficits are attributed to the chronic exposure of neutrophils to the inflammatory milieu of the wound, which induces a state of cellular senescence and exhaustion.^256^ Senescent neutrophils secrete proinflammatory mediators, such as IL‐1β and TNF‐α, and matrix‐degrading enzymes, like MMPs, which impair wound healing by promoting persistent inflammation and ECM degradation.^257^, ^258^ The impact of senescent neutrophils on wound healing has been demonstrated in both diabetic and nondiabetic ulcers.

Moreover, neutrophils in nonhealing wounds exhibit an increased propensity for NET formation, which can further exacerbate tissue damage and delay wound closure.^259^ NETs have been shown to trap and degrade growth factors, such as platelet‐derived growth factor (PDGF) and VEGF, impairing angiogenesis and granulation tissue formation.^260^ Targeting the molecular pathways that drive neutrophil dysfunction and NET formation, such as the PI3K–Akt axis and the NADPH oxidase complex, may offer therapeutic strategies to promote wound healing.^261^, ^262^

In COPD, a chronic inflammatory lung disease characterized by airflow obstruction and emphysema, neutrophils play a central role in the pathogenesis of the disease.^263^ Neutrophils in COPD exhibit a hyper‐activated phenotype, with increased production of ROS, proteases, and proinflammatory cytokines.^264^, ^265^ These mediators cause direct damage to the lung parenchyma, leading to alveolar destruction and airway remodeling.^266^ Additionally, neutrophils in COPD display an enhanced capacity for NET formation, which can further exacerbate lung injury and contribute to the development of autoimmunity.^267^ The role of neutrophil‐derived proteases and NETs in the progression of COPD has been highlighted in recent studies.

Recent studies have identified key molecular pathways that drive neutrophil plasticity in COPD. The activation of the NLRP3 inflammasome, a multiprotein complex that regulates the production of IL‐1β and IL‐18, has been implicated in the induction of a proinflammatory neutrophil phenotype.^268^ Furthermore, the PI3K–Akt–mTOR signaling axis has been shown to promote neutrophil survival and activation in COPD, contributing to the persistence of inflammation.^269^ Targeting these pathways may provide novel therapeutic approaches to modulate neutrophil plasticity and attenuate the progression of COPD. The potential of targeting the NLRP3 inflammasome and PI3K–Akt–mTOR signaling in COPD has been explored in recent studies.

In IBD, a group of chronic inflammatory disorders affecting the gastrointestinal tract, neutrophils have been implicated in the pathogenesis of the disease.^270^ Neutrophils in IBD exhibit a proinflammatory phenotype, with increased production of ROS, proteases, and cytokines that damage the intestinal epithelium and disrupt the mucosal barrier.^271^ Moreover, neutrophils in IBD display an enhanced capacity for NET formation, which can further exacerbate intestinal inflammation and promote the development of colorectal cancer.^272^ The contribution of neutrophil‐derived factors and NETs to the pathogenesis of IBD has been underscored by recent findings.

Recent studies have shed light on the molecular mechanisms driving neutrophil plasticity in IBD. The activation of the IL‐23/IL‐17 axis, a critical pathway in the pathogenesis of IBD, has been shown to promote a proinflammatory neutrophil phenotype.^273^ Additionally, the dysregulation of the Wnt/β‐catenin signaling pathway in neutrophils has been implicated in the impairment of intestinal wound healing and the perpetuation of inflammation.^274^ Targeting these pathways may offer therapeutic opportunities to modulate neutrophil plasticity and promote mucosal healing in IBD.^275^

### 5.3 Neutrophil plasticity in fibrotic diseases

Fibrotic diseases, characterized by the excessive deposition of ECM and the progressive loss of organ function, represent a significant healthcare burden worldwide.^276^ Recent studies have highlighted the critical role of neutrophil plasticity in the pathogenesis of various fibrotic conditions, including idiopathic pulmonary fibrosis (IPF), liver fibrosis, and systemic sclerosis (SSc).^2^, ^277^, ^278^

In IPF, a chronic and progressive interstitial lung disease, evidence suggests that neutrophils are implicated in the initiation and progression of fibrosis.^279^ Neutrophils in IPF exhibit a profibrotic phenotype characterized by the increased production of TGF‐β, a potent inducer of fibroblast activation and ECM deposition.^280^ Moreover, neutrophils in IPF display an enhanced capacity for NET formation, promoting the differentiation of fibroblasts into myofibroblasts and stimulating collagen production.^279^, ^281^

Recent studies have identified key molecular pathways that drive neutrophil plasticity in IPF. The activation of the NLRP3 inflammasome in neutrophils has been shown to promote the release of profibrotic mediators, such as IL‐1β and TGF‐β.^282^ Additionally, the PI3K–Akt signaling axis has been implicated in the induction of a profibrotic neutrophil phenotype, with studies demonstrating that inhibiting this pathway attenuates fibrosis in preclinical models of IPF.^283^

In liver fibrosis, a common outcome of chronic liver injuries, accumulating evidence suggests that neutrophils contribute to the initiation and progression of fibrosis.^284^ Neutrophils in liver fibrosis exhibit a profibrotic phenotype, with increased production of TGF‐β and PDGF, which promote the activation and proliferation of hepatic stellate cells (HSCs), the primary source of ECM in the fibrotic liver.^285^, ^286^ Moreover, neutrophils in liver fibrosis display an enhanced capacity for NET formation, stimulating the differentiation of HSCs into myofibroblasts and promoting collagen production.^287^

Recent studies have shed light on the molecular mechanisms driving neutrophil plasticity in liver fibrosis. The activation of the CXCR4/CXCL12 axis has been shown to promote the recruitment and activation of neutrophils in the fibrotic liver, contributing to the progression of fibrosis.^288^ Additionally, the dysregulation of the Hedgehog signaling pathway in neutrophils has been implicated in the induction of a profibrotic phenotype, with studies showing that inhibiting this pathway attenuates liver fibrosis in preclinical models.^289^

Emerging evidence implicates neutrophils in the pathogenesis of SSc, a chronic autoimmune disorder characterized by skin and organ fibrosis.^277^ Neutrophils in SSc exhibit a profibrotic phenotype, with increased production of TGF‐β and connective tissue growth factor, which promote the activation and differentiation of fibroblasts into myofibroblasts.^290^ Moreover, neutrophils in SSc display an enhanced capacity for NET formation, which can stimulate the production of autoantibodies and promote the activation of fibroblasts.^291^

Recent studies have identified key molecular pathways that drive neutrophil plasticity in SSc. The activation of the TLR4 signaling pathway in neutrophils has been shown to promote the release of profibrotic mediators, such as TGF‐β and IL‐6.^292^ Additionally, the dysregulation of the Wnt/β‐catenin signaling pathway in neutrophils has been implicated in the induction of a profibrotic phenotype, with studies demonstrating that the inhibition of this pathway attenuates skin fibrosis in preclinical models of SSc.^293^

**FIGURE 4:**
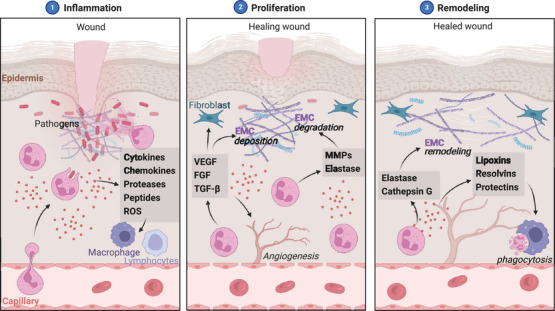
Neutrophil plasticity in tissue damage diseases. Excessive or dysregulated neutrophil activation contribute to the pathogenesis of various acute injuries, chronic injuries, and fibrotic diseases. Created with BioRender.com.

In conclusion, neutrophil plasticity plays a crucial role in the pathogenesis of various tissue damage diseases, including acute injuries, chronic injuries, and fibrotic conditions. Neutrophils exhibit remarkable phenotypic and functional adaptability in response to the microenvironmental cues encountered in these pathological states, acquiring distinct phenotypes that can either exacerbate tissue damage or promote tissue repair and resolution of inflammation. The molecular mechanisms driving neutrophil plasticity involve a complex interplay of signaling pathways, transcriptional regulators, and epigenetic modifications. These provide potential therapeutic targets for modulating neutrophil functions in tissue damage diseases (Figure 4).

### 5.4 Neutrophil plasticity in metabolic diseases

Emerging evidence suggests that neutrophils play a crucial role in the pathogenesis of metabolic diseases, such as nonalcoholic fatty liver disease (NAFLD) and its more severe form, NASH.^294^, ^295^ NASH is characterized by hepatic steatosis, inflammation, and progressive fibrosis, which can lead to cirrhosis and hepatocellular carcinoma.^296^, ^297^

Recent studies have highlighted the involvement of neutrophils in the progression of NAFLD to NASH. In the context of NASH, neutrophils exhibit a proinflammatory phenotype, with increased production of ROS, proteases, and proinflammatory cytokines, which contribute to hepatocyte damage and the perpetuation of inflammation.^298^ Moreover, neutrophils in NASH display an enhanced capacity for NET formation, which can further exacerbate liver injury and promote fibrosis.^295^

The plasticity of neutrophils in NASH is regulated by various factors, including lipid mediators, cytokines, and metabolic stress. Free fatty acids, particularly saturated fatty acids, have been shown to activate neutrophils through TLR4 signaling, inducing a proinflammatory phenotype.^299^ Additionally, the CXCR2 ligands CXCL1 and CXCL2, which are upregulated in the liver during NASH, promote neutrophil recruitment and activation.^300^, ^301^

Recent studies have identified key molecular pathways that drive neutrophil plasticity in NASH. The NLRP3 inflammasome, a multiprotein complex that regulates the production of IL‐1β and IL‐18, has been implicated in the induction of a proinflammatory neutrophil phenotype in NASH.^298^, ^302^ Furthermore, the receptor‐interacting protein kinase 3 (RIPK3), a key regulator of necroptosis, has been shown to promote neutrophil infiltration and activation in NASH.^303^

Targeting neutrophil plasticity in NASH represents a promising therapeutic strategy. Inhibition of CXCR2 signaling has been shown to attenuate neutrophil recruitment and ameliorate liver inflammation and fibrosis in preclinical models of NASH.^304^ Additionally, pharmacological inhibition of RIPK3 has been found to reduce neutrophil infiltration and improve liver function in experimental NASH.^303^

In conclusion, neutrophil plasticity plays a crucial role in the pathogenesis of metabolic diseases like NASH. Neutrophils acquire a proinflammatory and profibrotic phenotype in response to lipid mediators, cytokines, and metabolic stress, contributing to liver injury and fibrosis. Targeting the molecular pathways that drive neutrophil plasticity in NASH, such as CXCR2 and RIPK3 signaling, may offer novel therapeutic opportunities for the treatment of this prevalent and progressive metabolic disorder.

### 5.5 Neutrophil heterogeneity and plasticity in aging

Aging is associated with significant changes in the immune system, a phenomenon known as immunosenescence, which contributes to increased susceptibility to infections, reduced vaccine efficacy, and a higher incidence of chronic inflammatory diseases in the elderly population.^305^, ^306^ Neutrophils, as key players in innate immunity, undergo substantial alterations in their phenotype and function during the aging process, which may have important implications for age‐related immune dysfunction.^307^, ^308^

Recent studies have revealed that neutrophil heterogeneity and plasticity are significantly influenced by aging. Elderly individuals exhibit a higher proportion of immature neutrophils in the circulation, characterized by a banded nuclear morphology and reduced expression of surface markers like CD16.^309^ These immature neutrophils display impaired phagocytic capacity, reduced ROS production, and diminished chemotactic ability compared with their mature counterparts.^310^ The increased presence of immature neutrophils in the elderly may reflect a compensatory mechanism to maintain neutrophil numbers in the face of declining bone marrow function.^311^, ^312^

Moreover, aging is associated with the emergence of distinct neutrophil subsets with altered functional profiles. For instance, a subset of LDNs has been found to accumulate in the circulation of elderly individuals.^313^ These LDNs exhibit a proinflammatory phenotype, with increased production of cytokines like IL‐8 and TNF‐α, and enhanced NET formation.^314^ The expansion of proinflammatory neutrophil subsets in the elderly may contribute to the chronic low‐grade inflammation (inflammaging) that characterizes the aging process.

Neutrophil plasticity is also affected by aging, with elderly neutrophils displaying reduced responsiveness to microenvironmental cues and impaired ability to switch between proinflammatory and proresolving phenotypes.^315^ This loss of plasticity may hinder the resolution of inflammation and tissue repair in the elderly, leading to prolonged inflammatory responses and delayed wound healing.^316^

The molecular mechanisms underlying age‐related changes in neutrophil heterogeneity and plasticity are complex and multifaceted. Alterations in signaling pathways, such as decreased PI3K/Akt activation and increased p38 MAPK signaling, have been implicated in the functional deficits of elderly neutrophils.^317^, ^318^ Additionally, epigenetic modifications, such as changes in histone acetylation and DNA methylation patterns, may contribute to the altered transcriptional profiles and functional states of neutrophils during aging.^319^

Targeting age‐related changes in neutrophil heterogeneity and plasticity may offer novel therapeutic strategies for improving immune function and reducing the burden of age‐related diseases. For instance, interventions aimed at restoring the balance between immature and mature neutrophil subsets, such as G‐CSF administration or mTOR inhibition, have shown promise in enhancing neutrophil function in elderly individuals.^320^, ^321^ Moreover, modulating the polarization of neutrophils toward a proresolving phenotype, through the use of SPMs or other immunomodulatory agents, may promote the resolution of inflammation and tissue repair in the aging population.

### 5.6 Neutrophil heterogeneity and plasticity in tumor progression

Neutrophils play a complex and multifaceted role in the tumor microenvironment, exhibiting both protumorigenic and antitumorigenic functions depending on their phenotypic state and the specific context of the tumor.^77^, ^322^ The heterogeneity and plasticity of neutrophils in cancer have gained increasing attention in recent years, as they may offer novel targets for cancer immunotherapy.

TANs exhibit distinct phenotypes and functions compared with their circulating counterparts, reflecting the profound influence of the tumor microenvironment on neutrophil plasticity.^323^ TANs can be broadly classified into two subsets: N1 (antitumorigenic) and N2 (protumorigenic) neutrophils, which mirror the M1/M2 polarization paradigm of macrophages.^324^, ^325^ N1 neutrophils display an activated phenotype, with increased expression of proinflammatory cytokines, enhanced ROS production, and potent antitumor cytotoxicity.^326^ In contrast, N2 neutrophils exhibit an immunosuppressive and proangiogenic phenotype, characterized by the production of growth factors like VEGF and matrix‐degrading enzymes that promote tumor growth and metastasis.^327^

The polarization of TANs toward an N1 or N2 phenotype is regulated by a complex interplay of signals within the tumor microenvironment. Factors like TGF‐β, IL‐10, and G‐CSF have been shown to promote the N2 phenotype, while IFN‐γ and TNF‐α favor the N1 state.^328^ Moreover, the hypoxic and metabolically challenging conditions within the tumor can influence neutrophil plasticity, with HIF‐1α driving the acquisition of protumorigenic functions in TANs.^329^

In addition to the N1/N2 classification, recent single‐cell transcriptomic analyses have revealed further heterogeneity within the TAN population, identifying distinct subsets with unique gene expression profiles and functional properties.^330^, ^331^ For instance, a subset of TANs expressing high levels of programmed death‐ligand 1 (PD‐L1) has been identified in several cancer types, which may contribute to the immunosuppressive microenvironment and facilitate tumor immune escape.^332^, ^333^

The plasticity of TANs has important implications for cancer progression and response to therapy. The ability of neutrophils to switch between protumorigenic and antitumorigenic states in response to microenvironmental cues suggests that targeting the pathways that regulate neutrophil plasticity may offer a means to reprogram TANs toward an antitumor phenotype. For example, inhibiting TGF‐β signaling or enhancing IFN‐γ responses may promote the polarization of TANs toward an N1 state, enhancing their antitumor functions.^20^, ^334^

Moreover, the heterogeneity of TANs may influence the efficacy of cancer immunotherapies, such as immune checkpoint inhibitors. The presence of immunosuppressive neutrophil subsets, like PD‐L1‐expressing TANs, may limit the effectiveness of these therapies by inhibiting T cell responses.^335^, ^336^ Targeting these specific neutrophil subpopulations, in combination with existing immunotherapies, may enhance therapeutic outcomes in cancer patients.

In conclusion, neutrophil heterogeneity and plasticity play crucial roles in shaping the immune response in aging and cancer. Age‐related changes in neutrophil phenotypes and functions contribute to immunosenescence and the development of chronic inflammatory conditions in the elderly. In the context of cancer, the polarization of neutrophils toward protumorigenic or antitumorigenic states in response to microenvironmental cues has profound implications for tumor progression and response to therapy.^337^ Unraveling the molecular mechanisms that govern neutrophil heterogeneity and plasticity in these contexts may pave the way for novel therapeutic strategies aimed at harnessing the power of neutrophils to promote healthy aging and combat cancer.

## 6 ROLE OF NEUTROPHILS IN TISSUE REPAIR AND REGENERATION

Neutrophils have long been recognized as the first responders of the innate immune system, rapidly mobilized to sites of infection or injury to combat invading pathogens and initiate inflammatory responses. However, a growing body of evidence has challenged the traditional view of neutrophils as solely destructive cells, unveiling their multifaceted roles in orchestrating tissue repair and regenerative processes. This newfound appreciation of neutrophils as critical players in wound healing and tissue remodeling has profound implications for understanding physiological and pathological processes and developing novel therapeutic strategies.

### 6.1 Neutrophil‐mediated regulation of the wound healing cascade

The wound‐healing process is a highly coordinated and dynamic cascade involving multiple cellular and molecular events. Neutrophils are among the first immune cells to infiltrate the wound site, where they combat potential pathogens and actively regulate various stages of the wound‐healing cascade.^36^

In the initial inflammatory phase, neutrophils release many cytokines, chemokines, and proteolytic enzymes that shape the local microenvironment. These factors orchestrate the recruitment of additional immune cells, such as macrophages and lymphocytes, and initiate the clearance of cellular debris and foreign materials.^14^ Neutrophil‐derived antimicrobial peptides, such as the human cathelicidin LL‐37 and human α‐defensins, exhibit potent antimicrobial activities, modulate the inflammatory response, and promote angiogenesis, a crucial process for wound healing.^338^, ^339^

As the inflammatory phase subsides, neutrophils are pivotal in transitioning to the proliferative phase. By releasing matrix metalloproteinases (MMPs), including MMP‐2 and MMP‐9, and NE, neutrophils facilitate the degradation of the provisional ECM, creating a permissive environment for the migration and proliferation of endothelial cells, fibroblasts, and keratinocytes.^340^, ^341^ Concurrently, neutrophils produce various growth factors, such as VEGF, fibroblast growth factor (FGF), and TGF‐β, which stimulate angiogenesis, fibroblast activation, and ECM deposition.^342^, ^343^, ^344^

During the remodeling phase, neutrophils contribute to the resolution of inflammation and the maturation of the newly formed tissue. Neutrophil‐derived serine proteases, including NE and cathepsin G, participate in the degradation and remodeling of the ECM, particularly at the epidermal–dermal junction, facilitating the formation of a mature scar.^345^, ^346^ Additionally, neutrophils produce SPMs, such as lipoxins, resolvins, and protectins, which promote the clearance of apoptotic cells and the resolution of inflammation.^150^, ^151^

**FIGURE 5:**
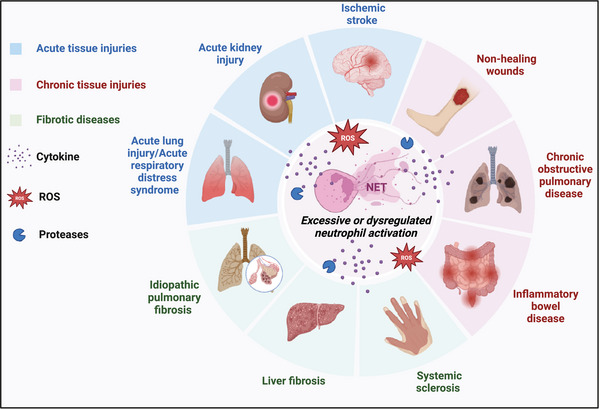
Neutrophil play crucial roles during wound healing. Neutrophils are recruited to the wound in the initial inflammatory phase. The recruited neutrophils release various cytokines, chemokines, and proteolytic enzymes that shape the local microenvironment, facilitating the removal of microbes, promoting angiogenesis, and contributing to the remodeling of newly formed tissue. Created with BioRender.com.

Recent studies have shed light on the dynamic phenotypic changes that neutrophils undergo during wound healing. In the early inflammatory phase, neutrophils exhibit a proinflammatory phenotype characterized by enhanced production of ROS, cytokines, and proteases.^122^ Moreover, neutrophils can form extracellular traps (NETs) that further contribute to bacterial killing and modulation of the inflammatory response.^347^ However, as the healing progresses, neutrophils can adopt an anti‐inflammatory and proresolving phenotype, marked by increased production of SPMs and anti‐inflammatory cytokines like IL‐10.^348^ This phenotypic plasticity enables neutrophils to orchestrate the transition from the inflammatory to the proliferative and remodeling phases, ensuring a coordinated and timely progression of the wound‐healing cascade^140^, ^179^ (Figure 5).

### 6.2 Neutrophil‐mediated regulation of angiogenesis and vascular remodeling

Angiogenesis, forming new blood vessels from pre‐existing vasculature, is a critical process in tissue repair and regeneration, ensuring the delivery of oxygen, nutrients, and immune cells to the site of injury or regeneration.^349^ Neutrophils have emerged as critical regulators of angiogenesis, exerting both proangiogenic and antiangiogenic effects depending on the context and their phenotypic state.^350^

In the early stages of wound healing, neutrophils promote angiogenesis by releasing various proangiogenic factors. Neutrophil‐derived VEGF, a potent inducer of endothelial cell proliferation and migration, is crucial in initiating the angiogenic response.^351^ Moreover, neutrophils secrete hepatocyte growth factor (HGF) and FGF‐2, which further stimulate endothelial cell proliferation and tube formation.^352^, ^353^ Additionally, neutrophils secrete matrix‐remodeling enzymes, such as MMPs and elastase, which facilitate the degradation of the basement membrane and ECM, creating a permissive environment for endothelial cell invasion and sprouting.^342^, ^354^

Neutrophils also contribute to the maturation and stabilization of newly formed blood vessels through the production of angiopoietins and the recruitment of pericytes and smooth muscle cells.^344^, ^355^ Neutrophil‐derived PDGF‐BB promotes pericyte recruitment and vessel stabilization.^356^ Furthermore, neutrophil‐derived SPMs, such as lipoxin A4 and resolvin D1, have enhanced endothelial cell proliferation, migration, and tube formation, supporting the later stages of angiogenesis.^357^, ^358^

Conversely, neutrophils can also exert antiangiogenic effects under certain conditions. During chronic inflammation or in the context of tumor angiogenesis, neutrophils can release factors that inhibit angiogenesis, such as angiostatin, endostatin, and thrombospondin‐1.^359^, ^360^, ^361^ NE has been shown to generate angiostatin from plasminogen, thus inhibiting angiogenesis.^359^ These antiangiogenic factors counteract the proangiogenic signals, limiting excessive vascular growth and promoting vascular normalization.^362^

The proangiogenic or antiangiogenic functions of neutrophils are influenced by their phenotypic state and the local microenvironment. Proinflammatory neutrophils, characterized by increased production of ROS and proinflammatory cytokines, tend to promote angiogenesis,^363^, ^364^ while anti‐inflammatory or proresolving neutrophils exhibit antiangiogenic properties.^20^, ^365^ The polarization of neutrophils toward a proangiogenic phenotype can be induced by hypoxia, growth factors, and cytokines present in the wound microenvironment.^21^, ^366^ This functional plasticity allows neutrophils to fine‐tune the angiogenic response during tissue repair and regeneration, ensuring an appropriate balance between vascular growth and resolution.^367^

### 6.3 Neutrophil‐mediated regulation of stem cell behavior and tissue regeneration

Emerging evidence suggests neutrophils are pivotal in regulating stem cell behavior and tissue regeneration processes.^368^, ^369^ By releasing various soluble factors and direct cell–cell interactions, neutrophils can modulate the proliferation, differentiation, and migration of stem and progenitor cells, thereby influencing tissue regeneration and homeostasis.^19^, ^355^ Neutrophils have been shown to regulate the behavior of various stem cell populations, including HSCs, mesenchymal stem cells, and tissue‐specific stem cells.

In the context of skeletal muscle regeneration, neutrophils have been shown to regulate the behavior of muscle stem cells, known as satellite cells.^370^ Neutrophil‐derived factors, such as VEGF, HGF, and LIF, promote satellite cells' activation, proliferation, and migration to the injury site.^371^, ^372^, ^373^ Additionally, neutrophils release proteases, including elastase and cathepsin G, which facilitate the degradation of the ECM and create a permissive environment for satellite cell migration and myofiber formation.^374^, ^375^ Neutrophil depletion has been shown to impair skeletal muscle regeneration, highlighting their crucial role in this process.^376^

In the liver, neutrophils play a crucial role in the regenerative response following injury or partial hepatectomy.^377^ Neutrophil‐derived cytokines, such as IL‐6 and TNF‐α, stimulate the proliferation of hepatocytes and liver progenitor cells, initiating the regenerative process.^378^, ^379^ Furthermore, neutrophils release proteases and ROS that activate MMPs, facilitating the remodeling of the ECM and creating a permissive environment for hepatocyte proliferation and migration.^380^ Neutrophil infiltration has been observed during the early stages of liver regeneration, and their depletion impairs the regenerative response.^381^

Neutrophils also contribute to intestinal epithelium regeneration following injury or inflammation.^382^ Neutrophil‐derived factors, such as HGF and GM‐CSF, promote the proliferation and migration of ISCs and their progeny.^383^ Additionally, neutrophils release antimicrobial peptides, such as α‐defensins, which exhibit antimicrobial properties and stimulate the proliferation and migration of intestinal epithelial cells, supporting epithelial regeneration.^384^, ^385^ Neutrophil infiltration is a hallmark of intestinal inflammation, and their presence has been associated with enhanced epithelial regeneration.^199^

The regulatory effects of neutrophils on stem cell behavior and tissue regeneration are not limited to the tissues above but extend to various other organs and systems, including the skin, lung, and nervous system.^386^ In these contexts, neutrophils modulate the behavior of resident stem and progenitor cells through the release of soluble factors, proteases, and direct cell–cell interactions, thereby influencing tissue homeostasis and regenerative processes.^387^ For example, in the skin, neutrophils promote wound healing by stimulating the proliferation and migration of epidermal stem cells and keratinocytes.^14^

### 6.4 Neutrophil‐mediated regulation of ECM remodeling

The ECM is a complex network of structural and functional proteins that provides structural support, regulates cell behavior, and serves as a reservoir for growth factors and cytokines.^388^ Neutrophils play a pivotal role in the dynamic remodeling of the ECM during tissue repair and regeneration, contributing to the degradation, deposition, and reorganization of ECM components.^386^

Neutrophils are a rich source of various proteolytic enzymes, including MMPs, serine proteases (such as NE and cathepsin G), and heparanase, which collectively degrade and remodel the ECM.^389^, ^390^ These enzymes facilitate the degradation of the provisional ECM, creating a permissive environment for cell migration, proliferation, and angiogenesis during the early stages of wound healing and tissue regeneration.^391^, ^392^

In addition to ECM degradation, neutrophils contribute to the deposition and reorganization of ECM components. Neutrophils release various ECM proteins, such as fibronectin, vitronectin, and tenascin‐C, which serve as scaffolds for cell migration and tissue remodeling.^393^, ^394^ Furthermore, neutrophils produce growth factors, such as TGF‐β and PDGF, which stimulate the production of ECM components by fibroblasts and other stromal cells.^395^

Endogenous inhibitors, such as TIMPs and α1‐antitrypsin, tightly regulate neutrophils' ECM‐remodeling activities.^396^ These inhibitors modulate the proteolytic activities of MMPs and serine proteases, respectively.^397^, ^398^ This fine‐tuned balance between ECM degradation and deposition is crucial for maintaining tissue homeostasis and preventing excessive tissue damage or fibrosis.^399^, ^400^

Recent studies have highlighted the role of neutrophil‐derived extracellular vesicles (EVs) in ECM remodeling.^401^, ^402^ Neutrophil EVs carry a cargo of proteolytic enzymes, growth factors, miRNAs,^403^ and signaling molecules that can modulate the behavior of target cells, such as fibroblasts and endothelial cells, and influence ECM remodeling processes.^404^ These EVs serve as vehicles for the intercellular transfer of bioactive molecules, enabling neutrophils to exert their regulatory effects on ECM remodeling and tissue repair in a paracrine manner.^403^, ^405^

### 6.5 Neutrophil‐mediated regulation of inflammation resolution and tissue homeostasis

While neutrophils are traditionally associated with initiating and propagating inflammatory responses, accumulating evidence suggests that they also play a crucial role in the resolution of inflammation and restoring tissue homeostasi.^140^, ^406^ This dual functionality of neutrophils is essential for maintaining a delicate balance between protective inflammatory responses and excessive tissue damage.^407^

During the resolution phase of inflammation, neutrophils undergo phenotypic and functional changes, transitioning from a proinflammatory state to an anti‐inflammatory and proresolving state.^164^ This transition is mediated by various endogenous and exogenous factors, including SPMs, such as lipoxins, resolvins, and protectins.^150^, ^408^ SPMs are derived from polyunsaturated fatty acids and are crucial in regulating the resolution of inflammation.^409^

Neutrophils are not only targets of SPMs but also active producers of these bioactive lipids.^409^ Through the enzymatic activity of LOXs and cyclooxygenases, neutrophils can synthesize SPMs from polyunsaturated fatty acids, such as arachidonic acid and docosahexaenoic acid.^410^ These SPMs exert potent anti‐inflammatory and proresolving effects by inhibiting neutrophil recruitment, promoting neutrophil apoptosis and efferocytosis (clearance of apoptotic cells), and stimulating the production of anti‐inflammatory cytokines like IL‐10.^411^, ^412^ SPMs also promote the polarization of macrophages toward an anti‐inflammatory phenotype.

In addition to SPMs, neutrophils contribute to the resolution of inflammation by producing other anti‐inflammatory mediators, such as annexin A1 and lactoferrin.^156^ Annexin A1, released by neutrophils during apoptosis, inhibits neutrophil recruitment and promotes macrophages’ clearance of apoptotic cells.^178^, ^413^ Lactoferrin, a multifunctional protein in neutrophil granules, exhibits anti‐inflammatory properties by inhibiting the production of proinflammatory cytokines and promoting the resolution of inflammation.^414^, ^415^ These mediators work in concert with SPMs to promote the resolution of inflammation.^178^

Furthermore, neutrophils play a crucial role in clearing apoptotic cells and cellular debris, a process essential for restoring tissue homeostasis.^164^, ^416^ Neutrophils can directly phagocytose apoptotic cells and cellular debris or release “find‐me” signals that attract professional phagocytes, such as macrophages, to the site of inflammation.^145^, ^416^ This clearance process is critical for preventing the release of intracellular contents and the perpetuation of inflammatory responses.^417^ Effective clearance of apoptotic cells by neutrophils and macrophages is essential for the resolution of inflammation and the prevention of autoimmunity.^416^

Recent studies have also highlighted the role of NETs in resolving inflammation While NETs are initially released as a defense mechanism against pathogens, they can also promote the resolution of inflammation by serving as a scaffold for the degradation of inflammatory mediators and the clearance of apoptotic cells.^418^, ^419^ However, excessive or dysregulated NET formation can contribute to tissue damage and the perpetuation of inflammatory responses, underscoring the importance of tightly regulating this proces.^123^, ^420^ The balance between the beneficial and detrimental effects of NETs is crucial for maintaining tissue homeostasis and preventing chronic inflammation.

In summary, neutrophils play multifaceted roles in tissue repair and regeneration, extending far beyond their traditional antimicrobial functions. Through the release of various soluble factors, proteolytic enzymes, and direct cell–cell interactions, neutrophils orchestrate the wound healing cascade, regulate angiogenesis and vascular remodeling, modulate stem cell behavior and tissue regeneration, and contribute to the dynamic remodeling of the ECM. Moreover, neutrophils actively participate in the resolution of inflammation and the restoration of tissue homeostasis, highlighting their versatility and plasticity in adapting to diverse microenvironmental cues. This newfound appreciation of neutrophils as critical players in tissue repair and regeneration opens up exciting avenues for developing novel therapeutic strategies targeting neutrophil functions in various pathological conditions, including chronic inflammatory disorders, tissue injury, and regenerative medicine.

## 7 THERAPEUTIC TARGETING OF NEUTROPHILS

The remarkable functional versatility and plasticity of neutrophils, enabling their transition between distinct phenotypic states, have profound implications for therapeutic intervention. As our understanding of neutrophil heterogeneity and their diverse roles in health and disease continues to evolve, new opportunities have emerged for selectively modulating neutrophil responses to mitigate detrimental inflammation or harness their reparative potential. This section explores the latest advances in therapeutic strategies targeting neutrophils, spanning small molecule inhibitors, biologics, cell‐based therapies, and nanomedicine platforms. We highlight disease‐specific treatment protocols and the diverse arsenal of drugs, materials, and delivery systems leveraged to modulate neutrophil phenotypes and functions.

### 7.1 Small molecule inhibitors

Small molecule inhibitors have been extensively explored for modulating neutrophil responses by targeting key signaling pathways, enzymes, and effector mechanisms. These compounds offer the advantages of high specificity, tunability, and potential for oral administration.

#### 7.1.1 Targeting inflammatory signaling pathways

Inhibitors of proinflammatory signaling cascades like NF‐κB, p38 MAPK, and PI3K/Akt have shown promise in attenuating excessive neutrophil activation and promoting proresolving phenotypes. For instance, the p38 MAPK inhibitor SB203580 has been shown to reduce neutrophil recruitment, oxidative burst, and cytokine production in preclinical models of ALI and rheumatoid arthritis.^421^, ^422^ Similarly, the PI3Kγ inhibitor AS605240 has demonstrated efficacy in mitigating neutrophil‐mediated inflammation and tissue damage in ischemia–reperfusion injury and autoimmune vasculitis.^423^, ^424^

#### 7.1.2 Inhibiting neutrophil effector mechanisms

Selective inhibition of neutrophil effector mechanisms like oxidative burst, degranulation, and NET formation represents another therapeutic strategy. The NADPH oxidase inhibitor apocynin has shown promise in reducing neutrophil‐mediated oxidative damage and inflammation in preclinical models of ARDS and IBD.^425^, ^426^ Additionally, inhibitors of PAD4, such as Cl‐amidine, have been explored for their ability to block NET formation and mitigate associated tissue damage in autoimmune disorders like rheumatoid arthritis and SLE.^427^, ^428^

#### 7.1.3 Modulating neutrophil survival and apoptosis

Compounds that modulate neutrophil lifespan and apoptosis pathways have also garnered interest as therapeutic agents. The Bcl‐2 family inhibitor ABT‐737 has been shown to promote neutrophil apoptosis, reducing accumulation and associated tissue damage in preclinical models of ALI and ischemia–reperfusion injury.^429^ Conversely, inhibitors of caspase and proapoptotic pathways, like the pan‐caspase inhibitor Q‐VD‐OPh, have been explored to prolong neutrophil survival and enhance their antimicrobial functions in severe infections.^430^

#### 7.1.4 Targeting neutrophil exocytosis

Inhibitors of neutrophil exocytosis, such as Nexinhibs and SNARE domain‐derived peptide aptamers, have emerged as promising strategies to selectively modulate neutrophil degranulation without affecting other functions like phagocytosis. Nexinhibs interrupt the Rab27a–JFC1 interaction, selectively inhibiting azurophil granule release, while SNARE‐mimicking peptide aptamers exhibit varying selectivity toward different granule subsets.^431^, ^432^ These inhibitors have shown efficacy in attenuating neutrophil‐mediated inflammation and tissue damage in preclinical models of systemic inflammation, ALI, and sepsis.^433^

### 7.2 Biologics and targeted therapies

Biologic agents, including monoclonal antibodies, cytokines, and receptor antagonists, have emerged as powerful tools for selectively targeting neutrophil subpopulations or modulating specific neutrophil functions.^434^

#### 7.2.1 Targeting neutrophil surface receptors

Monoclonal antibodies and receptor antagonists have been developed to block key neutrophil surface receptors involved in recruitment, activation, and effector functions. For instance, the CXCR2 antagonist navarixin has shown promise in reducing neutrophil infiltration and associated tissue damage in preclinical models of ARDS, IBD, and ischemia–reperfusion injury.^435^, ^436^, ^437^ Similarly, the anti‐CD177 monoclonal antibody Hu‐AMLX7909 has been explored for its ability to selectively deplete proinflammatory low‐density granulocytes (LDGs) in autoimmune disorders like ANCA‐associated vasculitis.^438^

#### 7.2.2 Modulating neutrophil phenotypes with cytokines

Cytokines and growth factors have been leveraged to promote the polarization of neutrophils toward proresolving or proinflammatory phenotypes.^27^, ^439^ For example, administration of IL‐10 has been shown to induce an N2‐like phenotype in neutrophils, enhancing their proresolving and tissue‐reparative functions in preclinical models of ALI and myocardial infarction.^440^, ^441^ Conversely, G‐CSF has been explored for its ability to mobilize and activate neutrophils, enhancing their antimicrobial functions in the context of severe infections and cancer immunotherapy.^442^, ^443^

#### 7.2.3 Targeting neutrophil‐derived mediators

Biologic agents have also been developed to neutralize or modulate the activity of neutrophil‐derived mediators implicated in disease pathogenesis. For instance, the anticalprotectin antibody ABX‐CBL has been explored for its ability to neutralize the proinflammatory effects of calprotectin released by neutrophils in IBD and rheumatoid arthritis.^444^, ^445^ Similarly, the DNase I enzyme dornase alfa has been investigated for its ability to degrade NETs and mitigate associated tissue damage in conditions like cystic fibrosis and COVID‐19.^446^, ^447^

#### 7.2.4 Targeting NETs

Given the pathogenic role of NETs in various inflammatory and autoimmune disorders, strategies targeting NET formation or promoting NET clearance have gained significant attention. Biologic agents like monoclonal antibodies against NET components (e.g., antihistone antibodies) or enzymes involved in NET formation (e.g., NE inhibitors) have shown promise in preclinical models.^448^, ^449^ Additionally, the use of DNase I to degrade NETs has been explored in clinical trials for conditions like COVID‐19‐associated ARDS and cystic fibrosis.^450^

### 7.3 Cell‐based therapies

Cell‐based therapies have emerged as a promising approach for treating various diseases, including cancer, inflammatory disorders, and wound healing. Neutrophils, the most abundant type of white blood cells, have gained increasing attention as potential therapeutic agents due to their unique functions and abilities to target specific sites of inflammation or injury.^451^ Neutrophils possess a remarkable capacity to migrate to sites of inflammation, where they release a variety of bioactive molecules, such as cytokines, chemokines, and growth factors, which can modulate the immune response and promote tissue repair.^452^

Recent studies have explored the potential of using neutrophils as drug delivery vehicles for targeted therapy.^9^ By exploiting the natural homing ability of neutrophils, researchers have developed strategies to load neutrophils with therapeutic agents, such as drugs, nanoparticles, or genetic material, which can then be delivered to specific sites of interest. For example, neutrophils have been used to deliver anticancer drugs to tumor sites, resulting in enhanced therapeutic efficacy and reduced systemic side effects.^453^

In addition to their role as drug carriers, neutrophils have also been found to release EVs, which can mediate intercellular communication and modulate the immune response. Neutrophil‐derived EVs have been shown to contain a variety of bioactive molecules, such as proteins, lipids, and nucleic acids, which can influence the behavior of recipient cells.^405^ For instance, neutrophil‐derived EVs have been found to promote the proliferation and migration of breast cancer cells by releasing oncogenic miRNAs.

The ability to reprogram neutrophils has also opened up new possibilities for cell‐based therapies. By modulating the phenotype and function of neutrophils, researchers aim to enhance their therapeutic potential and minimize potential side effects. For example, neutrophils have been shown to undergo temporal polarization following myocardial infarction, which can influence their role in tissue repair and regeneration.^454^

The use of nanoparticles has further expanded the therapeutic potential of neutrophils. Nanoparticles can be designed to target specific receptors or pathways in neutrophils, enabling the modulation of their function and behavior. For instance, nanoparticles coated with neutrophil membranes have been shown to effectively treat cancer metastasis by mimicking the natural targeting ability of neutrophils.^454^

Despite the promising potential of neutrophil‐based therapies, several challenges remain to be addressed. One of the main concerns is the potential off‐target effects of neutrophils, which can lead to tissue damage and exacerbate inflammation.^266^ Additionally, the short lifespan of neutrophils and their sensitivity to environmental factors can limit their therapeutic efficacy.^455^

To overcome these challenges, researchers are exploring various strategies to enhance the stability and functionality of neutrophils. For example, genetic engineering approaches have been used to modify neutrophils to express specific receptors or proteins that can enhance their targeting ability or prolong their lifespan.^456^ Additionally, the use of biomaterials, such as hydrogels or scaffolds, can provide a supportive microenvironment for neutrophils, enabling their sustained release and function at the target site.^416^

### 7.4 Nanomedicine platforms

Nanotechnology has emerged as a powerful tool for modulating neutrophil responses and enabling targeted delivery of therapeutic payloads. Nanoparticles can be engineered with specific surface modifications, drug payloads, and imaging capabilities to target neutrophils or selectively modulate their phenotypes and functions.^9^, ^457^

#### 7.4.1 Neutrophil‐targeted nanoparticles

Nanoparticles can be functionalized with neutrophil‐specific targeting ligands, such as antibodies or peptides, to enable selective binding and neutrophil uptake.^456^ This approach has been explored for delivering therapeutic payloads, including small molecule inhibitors, siRNAs, and cytotoxic agents, to modulate neutrophil responses or selectively deplete pathogenic neutrophil subpopulations.^9^ For instance, neutrophil‐targeted nanoparticles loaded with the p38 MAPK inhibitor SB239063 have attenuated neutrophil‐mediated inflammation and tissue damage in preclinical acute lung and ischemia–reperfusion injury models.^458^

#### 7.4.2 Neutrophil membrane‐coated nanoparticles

Leveraging the unique properties of neutrophil cell membranes, nanoparticles can be coated with neutrophil‐derived membranes to enhance their biocompatibility, circulation time, and targeting capabilities.^459^ These biomimetic nanoparticles can be loaded with therapeutic payloads and have shown promise in delivering anti‐inflammatory agents or cytotoxic drugs to sites of inflammation or tumors, exploiting the innate homing abilities of neutrophils.^453^

#### 7.4.3 Nanoparticles for modulating neutrophil phenotypes

Nanoparticles can be engineered to modulate neutrophil phenotypes and functions by delivering specific payloads or interacting with key signaling pathways. For instance, nanoparticles loaded with resolvins or other SPMs have been explored for their ability to reprogram neutrophils toward proresolving phenotypes, enhancing tissue repair and resolution of inflammation.^452^ Additionally, nanoparticles functionalized with specific ligands or antibodies have been investigated for their ability to selectively activate or inhibit neutrophil surface receptors, modulating their recruitment, activation, and effector functions.^9^

#### 7.4.4 Nanoparticle‐mediated delivery across biological barriers

Nanoparticles can be designed to exploit neutrophils’ ability to cross biological barriers, such as the blood–brain barrier or the endothelium, enabling targeted delivery of therapeutic payloads to otherwise inaccessible sites. For instance, neutrophil‐mediated delivery of drug‐loaded albumin nanoparticles has effectively crossed the blood vessel barrier and mitigated inflammation in preclinical models of lipopolysaccharide (LPS)‐induced inflammation and Pseudomonas aeruginosa infection.^458^

### 7.5 Disease‐specific treatment protocols

The therapeutic targeting of neutrophils has been explored across various disease contexts, with diverse treatment protocols and strategies tailored to specific pathological mechanisms and clinical needs.^1^, ^2^, ^3^

#### 7.5.1 Acute inflammatory disorders

In conditions like ARDS, sepsis, and ischemia–reperfusion injury, therapeutic strategies have focused on attenuating excessive neutrophil activation and associated tissue damage.^460^, ^461^ Treatment protocols may involve the administration of small molecule inhibitors targeting inflammatory signaling pathways (e.g., p38 MAPK inhibitors), oxidative burst inhibitors (e.g., NADPH oxidase inhibitors), or agents that modulate neutrophil lifespan and apoptosis (e.g., Bcl‐2 family inhibitors).^318^, ^462^, ^463^

#### 7.5.2 Chronic inflammatory and autoimmune disorders

In conditions like rheumatoid arthritis, SLE, and ANCA‐associated vasculitis, therapeutic strategies have aimed to target pathogenic neutrophil subpopulations or modulate their proinflammatory phenotypes selectively.^464^, ^465^ Treatment protocols may involve the administration of monoclonal antibodies targeting specific neutrophil surface markers (e.g., anti‐CD177 antibodies for depleting LDGs) or small molecule inhibitors targeting neutrophil effector mechanisms like NET formation (e.g., PAD4 inhibitors).^11^

#### 7.5.3 Impaired wound healing and fibrosis

In conditions characterized by impaired wound healing, such as chronic nonhealing wounds or excessive fibrosis, therapeutic strategies have aimed to promote neutrophils’ proresolving and tissue‐reparative functions.^466^ Treatment protocols may involve the administration of SPMs or cytokines like IL‐10 to induce proresolving neutrophil phenotypes.^467^

#### 7.5.4 Infectious diseases

In the context of severe infections or immunodeficiencies, therapeutic strategies have focused on enhancing the antimicrobial functions of neutrophils or prolonging their lifespan.^468^, ^469^ Treatment protocols may involve administering cytokines like G‐CSF to mobilize and activate neutrophils or using caspase inhibitors or antiapoptotic agents to prolong neutrophil survival and enhance their antimicrobial functions^470^ (Table 4).

**TABLE 4:** Therapeutic targeting of neutrophils.

Types	Targets	Molecules/materials	Description
Small molecule inhibitors	‐ Inflammatory signaling pathways (NF‐κB, p38 MAPK, PI3K/Akt)^421^, ^422^, ^423^, ^424^	‐ SB203580, AS605240^421^, ^422^, ^423^	‐ Attenuate excessive neutrophil activation and promote proresolving phenotypes^421^, ^422^, ^423^, ^424^
	‐ Neutrophil effector mechanisms (oxidative burst, degranulation, NET formation)^395^, ^425^, ^427^, ^428^	‐ Apocynin, Cl‐amidine^395^, ^425^, ^427^, ^428^	‐ Reduce neutrophil‐mediated oxidative damage, inflammation, and tissue damage^395^, ^425^, ^427^, ^428^
	‐ Neutrophil survival and apoptosis^429^, ^430^	‐ ABT‐737, Q‐VD‐OPh^429^, ^430^	‐ Modulate neutrophil lifespan to reduce accumulation and associated tissue damage or enhance antimicrobial function^429^, ^430^
	‐ Neutrophil exocytosis^431^, ^432^, ^433^	‐ Nexinhibs, SNARE domain‐derived peptide aptamers^431^, ^432^, ^433^	‐ Selectively modulate neutrophil degranulation without affecting other functions^431^, ^432^, ^433^
Biologic molecules	‐ Neutrophil surface receptors^435^, ^436^, ^437^, ^438^	‐ Navarixin, Hu‐AMLX7909^435^, ^436^, ^437^, ^438^	‐ Block key receptors involved in recruitment, activation, and effector functions or deplete proinflammatory subsets^435^, ^436^, ^437^, ^438^
	‐ Neutrophil phenotypes^27^, ^439^, ^440^, ^441^, ^442^, ^443^	‐ IL‐10, G‐CSF^27^, ^439^, ^440^, ^441^, ^442^, ^443^	‐ Promote polarization toward proresolving or proinflammatory phenotypes^27^, ^439^, ^440^, ^441^, ^442^, ^443^
	‐ Neutrophil‐derived mediators^444^, ^445^, ^446^, ^447^	‐ ABX‐CBL, dornase alfa^444^, ^445^, ^446^, ^447^	‐ Neutralize or modulate the activity of mediators implicated in disease pathogenesis^444^, ^445^, ^446^, ^447^
	‐ NETs^448^, ^449^, ^450^	‐ Antihistone antibodies, neutrophil elastase inhibitors, DNase I ^448^, ^449^, ^450^	‐ Target NET formation or promote NET clearance^448^, ^449^, ^450^
Cell‐based therapies	‐ Neutrophils as drug delivery vehicles^9^, ^453^	‐ Drug‐loaded neutrophils ^9^, ^453^	‐ Exploit the natural homing ability of neutrophils for targeted delivery of therapeutic agents^9^, ^453^
	‐ Neutrophil‐derived extracellular vesicles (EVs)^450^, ^471^, ^472^	‐ Neutrophil‐derived EVs^450^, ^471^, ^472^	‐ Mediate intercellular communication and modulate the immune response^450^, ^471^, ^472^
	‐ Reprogramming neutrophils^473^, ^474^	‐ Modulated neutrophils^473^, ^474^	‐ Enhance therapeutic potential and minimize side effects by modulating phenotype and function^473^, ^474^
	‐ Neutrophil–nanoparticle interactions^475^, ^476^	‐ Neutrophil membrane‐coated nanoparticles^475^, ^476^	‐ Target specific receptors or pathways in neutrophils to modulate function and behavior^475^, ^476^
Nanomedicine platforms	‐ Neutrophil‐targeted nanoparticles^453^, ^458^, ^477^, ^478^	‐ Ligand‐functionalized nanoparticles^453^, ^458^, ^477^, ^478^	‐ Enable selective binding, uptake, and modulation of neutrophil responses^453^, ^458^, ^477^, ^478^
	‐ Neutrophil membrane‐coated nanoparticles^453^, ^459^, ^479^	‐ Neutrophil membrane‐coated nanoparticles^453^, ^459^, ^479^	‐ Enhance biocompatibility, circulation time, and targeting capabilities^453^, ^459^, ^479^
	‐ Nanoparticles for modulating neutrophil phenotypes^452^, ^480^	‐ Resolvin‐loaded nanoparticles, ligand‐functionalized nanoparticles ^452^, ^480^	‐ Reprogram neutrophils toward proresolving phenotypes or modulate surface receptor activity ^452^, ^480^
	‐ Nanoparticle‐mediated delivery across biological barriers^458^, ^481^, ^482^, ^483^	‐ Neutrophil‐mediated nanoparticle delivery^458^, ^481^, ^482^, ^483^	‐ Exploit neutrophils' ability to cross biological barriers for targeted delivery of therapeutic payloads^458^, ^481^, ^482^, ^483^

Abbreviations: MAPK, mitogen‐activated protein kinase; G‐CSF, granulocyte colony‐stimulating factor; NETs, neutrophil extracellular traps.

### 7.6 Preclinical and clinical studies on neutrophil‐targeted therapies

The growing understanding of neutrophil biology and its role in various pathological conditions has led to an increasing number of preclinical studies and clinical trials aimed at modulating neutrophil functions for therapeutic benefit. This section provides an overview of key preclinical animal experiments and clinical trials targeting neutrophils, organized by the main pathways and mechanisms of action.

#### 7.6.1 Targeting inflammatory signaling pathways

##### p38 MAPK inhibition

Preclinical studies have demonstrated the potential of p38 MAPK inhibitors in attenuating neutrophil‐mediated inflammation and tissue damage. For instance, the p38 MAPK inhibitor SB203580 has shown efficacy in reducing neutrophil recruitment, oxidative burst, and cytokine production in animal models of ALI and rheumatoid arthritis.^421^, ^422^, ^484^ In a mouse model of ALI induced by LPS, treatment with SB203580 significantly reduced neutrophil infiltration into the lungs, decreased levels of proinflammatory cytokines (TNF‐α, IL‐1β), and improved overall lung function.^421^ Similarly, in a rat model of collagen‐induced arthritis, SB203580 administration reduced joint swelling, synovial inflammation, and cartilage destruction, accompanied by decreased neutrophil activation and cytokine production.^422^, ^484^ Clinical trials have also explored p38 MAPK inhibitors in inflammatory conditions. A phase II randomized controlled trial (NCT00303563) evaluated the efficacy of the p38 MAPK inhibitor PH‐797804 in patients with COPD. The study demonstrated improvements in lung function and reduced systemic inflammation markers, although neutrophil‐specific outcomes were not reported.^485^

##### PI3K/Akt pathway inhibition

The PI3K/Akt signaling pathway is crucial in neutrophil activation and survival. Preclinical studies have shown promising results with PI3K inhibitors in various inflammatory conditions. In a mouse model of ischemia–reperfusion injury, the PI3Kγ inhibitor AS605240 significantly reduced neutrophil infiltration, decreased proinflammatory cytokine production, and attenuated tissue damage.^486^, ^487^ Another study using a rat model of autoimmune vasculitis demonstrated that AS605240 treatment reduced neutrophil accumulation in affected tissues and improved overall disease outcomes.^487^, ^488^ Clinical development of PI3K inhibitors for neutrophil‐mediated diseases is ongoing. A phase II clinical trial (NCT01462617) evaluated the PI3Kδ inhibitor idelalisib in patients with allergic rhinitis. While the primary focus was on B and T cell modulation, the study also assessed effects on neutrophil function, showing a reduction in neutrophil activation markers.

#### 7.6.2 Modulating neutrophil effector mechanisms

##### NADPH oxidase inhibition

Targeting the NADPH oxidase complex, responsible for neutrophil oxidative burst, has been explored in various inflammatory conditions. The NADPH oxidase inhibitor apocynin has shown efficacy in preclinical ARDS and IBD models. In a mouse model of LPS‐induced ARDS, apocynin treatment significantly reduced neutrophil infiltration decreased oxidative stress markers, and improved lung function.^489^, ^490^ Similarly, in a rat model of dextran sulfate sodium (DSS)‐induced colitis, apocynin administration attenuated neutrophil recruitment to the colon, reduced oxidative damage, and improved disease severity scores.^491^, ^492^ Clinical trials investigating NADPH oxidase inhibitors are limited, but some studies have explored their potential. A small pilot study (NCT02594371) evaluated the effects of apocynin inhalation in patients with mild asthma, showing a trend toward reduced airway neutrophilia and improved lung function, although larger trials are needed to confirm these findings.^493^

##### NET formation inhibition

NETs have been implicated in the pathogenesis of various inflammatory and autoimmune disorders. Inhibitors of peptidylarginine deiminase 4 (PAD4), a key enzyme in NET formation, have shown promise in preclinical studies. In a mouse model of collagen‐induced arthritis, treatment with the PAD4 inhibitor Cl‐amidine reduced NET formation, attenuated joint inflammation, and improved overall disease scores.^494^ Another study using a mouse model of lupus nephritis demonstrated that Cl‐amidine treatment decreased NET formation in the kidneys, reduced autoantibody production, and improved renal function.^427^, ^492^ Clinical trials targeting NET formation are in the early stages. A phase I/II clinical trial (NCT03910543) is currently evaluating the safety and efficacy of the PAD4 inhibitor GSK3145095 in patients with rheumatoid arthritis, with NET formation as a secondary outcome measure.^495^

#### 7.6.3 Modulating neutrophil survival and apoptosis

##### Bcl‐2 family inhibition

Targeting neutrophil survival pathways, particularly the Bcl‐2 family of proteins, has been explored as a strategy to promote neutrophil apoptosis and resolution of inflammation. In a mouse model of LPS‐induced ALI, treatment with the Bcl‐2 family inhibitor ABT‐737 enhanced neutrophil apoptosis, reduced neutrophil accumulation in the lungs, and improved overall lung function.^496^ Similarly, in a rat model of renal ischemia–reperfusion injury, ABT‐737 administration promoted neutrophil apoptosis, decreased neutrophil‐mediated tissue damage, and improved renal function.^497^, ^498^ Clinical development of Bcl‐2 family inhibitors for neutrophil‐mediated diseases is limited, with most studies focusing on cancer applications. However, a phase I clinical trial (NCT02391480) evaluated the effects of the Bcl‐2 inhibitor venetoclax on immune cell populations, including neutrophils, in healthy volunteers, providing insights into the potential modulation of neutrophil survival in vivo.^499^

##### Caspase inhibition

In contrast to promoting apoptosis, inhibiting caspase pathways has been explored as a strategy to prolong neutrophil survival and enhance antimicrobial functions in severe infections. A preclinical study using a mouse model of MRSA skin infection demonstrated that treatment with the pan‐caspase inhibitor Q‐VD‐OPh prolonged neutrophil survival, enhanced bacterial clearance, and improved overall survival rates.^500^, ^501^ Clinical trials specifically targeting neutrophil survival through caspase inhibition are limited. However, a phase II clinical trial (NCT01756105) evaluated the pan‐caspase inhibitor emricasan in patients with NASH, showing reductions in liver inflammation and fibrosis, although neutrophil‐specific outcomes were not reported.^502^

#### 7.6.4 Targeting neutrophil recruitment and trafficking

##### CXCR2 antagonism

Inhibition of the chemokine receptor CXCR2 has been explored as a strategy to reduce neutrophil recruitment to sites of inflammation. In a mouse model of ventilator‐induced lung injury, treatment with the CXCR2 antagonist danirixin significantly reduced neutrophil infiltration into the lungs, decreased levels of proinflammatory cytokines, and improved lung function.^503^ Another study using a mouse model of DSS‐induced colitis demonstrated that danirixin administration attenuated neutrophil recruitment to the colon and reduced overall disease severity.^504^ Clinical trials have also evaluated CXCR2 antagonists in various inflammatory conditions. A phase II randomized controlled trial (NCT01704495) assessed the efficacy of the CXCR2 antagonist navarixin in patients with COPD, showing reductions in sputum neutrophil counts and trends toward improved clinical outcomes.^505^, ^506^

#### 7.6.5 Nanoparticle‐based approaches

Nanoparticle‐based strategies for modulating neutrophil functions have gained increasing attention in recent years. A preclinical study using a mouse model of ALI demonstrated that neutrophil‐targeted nanoparticles loaded with the p38 MAPK inhibitor SB239063 effectively reduced neutrophil infiltration, decreased proinflammatory cytokine levels, and improved lung function compared with free drug administration.^507^, ^508^ Another study explored the use of neutrophil membrane‐coated nanoparticles for targeted drug delivery in a mouse model of inflammatory arthritis. These biomimetic nanoparticles, loaded with the anti‐inflammatory drug celastrol, showed enhanced accumulation in inflamed joints, reduced neutrophil infiltration, and improved clinical outcomes compared with free drug or conventional nanoparticles.^459^, ^509^ Clinical translation of nanoparticle‐based approaches for neutrophil modulation is still in early stages, with most studies currently in preclinical development (Table 5).

**TABLE 5:** Summary of key preclinical and clinical studies targeting neutrophils.

Therapeutic target	Preclinical studies	Clinical trials
p38 MAPK	SB203580 reduced neutrophil recruitment and inflammation in acute lung injury^421^ and rheumatoid arthritis^484^ models.	Phase II trial of PH‐797804 (NCT00303563) in COPD showed improved lung function and reduced systemic inflammation.
PI3K/Akt	AS605240 reduced neutrophil infiltration and inflammation in ischemia–reperfusion injury^510^ and autoimmune vasculitis^487^ models.	Phase II trial of idelalisib (NCT01462617) in allergic rhinitis assessed effects on neutrophil activation.
NADPH Oxidase	Apocynin reduced neutrophil infiltration and inflammation in ARDS^490^ and IBD^511^ models.	Pilot study of inhaled apocynin (NCT02594371) in mild asthma showed trends of reduced airway neutrophilia.
PAD4 (NETs)	Cl‐amidine reduced NET formation and inflammation in arthritis494 and lupus nephritis^492^ models.	Phase I/II trial of GSK3145095 (NCT03910543) in rheumatoid arthritis with NET formation as secondary outcome.
Bcl‐2 Family	ABT‐737 enhanced neutrophil apoptosis and reduced inflammation in acute lung injury496 and renal ischemia–reperfusion^498^ models.	Phase I trial of venetoclax (NCT02391480) evaluated effects on immune cells, including neutrophils, in healthy subjects.
Caspases	Q‐VD‐OPh prolonged neutrophil survival and enhanced bacterial clearance in MRSA skin infection model.^501^	Phase II trial of emricasan (NCT01756105) in NASH showed reduced liver inflammation and fibrosis.
CXCR2	Danirixin reduced neutrophil infiltration and inflammation in ventilator‐induced lung injury^503^ and DSS‐induced colitis^504^ models.	Phase II trial of navarixin (NCT01704495) in COPD showed reduced sputum neutrophils and trends toward improved outcomes.
Nanoparticles	Neutrophil‐targeted nanoparticles with p38 inhibitor^508^ or neutrophil membrane‐coated nanoparticles with celastrol^509^ showed enhanced efficacy in preclinical models.	Clinical translation still in early preclinical stages.

*Data sources*: ClinicalTrials.gov.

Abbreviations: ARDS, acute respiratory distress syndrome; IBD, inflammatory bowel disease; MRSA, methicillin‐resistant staphylococcus aureus; COPD, chronic obstructive pulmonary disease.

## 8 CONCLUSION AND FUTURE PERSPECTIVES

The past decade has witnessed a paradigm shift in our understanding of neutrophil biology, unveiling a complex landscape of heterogeneity and plasticity that challenges the traditional view of neutrophils as a homogeneous population with limited functional capabilities. This evolution in our knowledge has profound implications for both basic immunology and translational medicine, opening new avenues for therapeutic interventions across a wide spectrum of diseases.

### 8.1 Key advances and their implications

#### 8.1.1 Neutrophil heterogeneity

The identification of distinct neutrophil subsets with diverse phenotypes and functions has revolutionized our understanding of neutrophil biology. This heterogeneity spans various dimensions, including maturation states, density, surface marker expression, and functional polarization. The recognition of specialized neutrophil populations, such as TANs or LDGs, has important implications for disease pathogenesis and potential targeted therapies.

#### 8.1.2 Neutrophil plasticity

The remarkable ability of neutrophils to adapt their transcriptional programs and functional states in response to microenvironmental cues has expanded our view of their roles in health and disease. This plasticity enables neutrophils to transition between proinflammatory and proresolving phenotypes, contributing to both the initiation and resolution of inflammation.

#### 8.1.3 Expanded functional repertoire

Beyond their canonical antimicrobial functions, neutrophils have emerged as critical players in diverse physiological and pathological processes, including tissue repair, angiogenesis, metabolism, and cancer progression. This expanded functional repertoire highlights the potential for neutrophil‐targeted therapies in a broader range of diseases than previously anticipated.

#### 8.1.4 Signaling networks

The elucidation of complex signaling networks governing neutrophil functions has provided new targets for therapeutic intervention. From recruitment and migration to antimicrobial activities and cell fate decisions, our understanding of these molecular pathways offers opportunities for fine‐tuning neutrophil responses in various disease contexts.

#### 8.1.5 Therapeutic approaches

The development of targeted therapies, including small molecule inhibitors, biologics, cell‐based therapies, and nanomedicine platforms, has opened new possibilities for modulating neutrophil functions in disease. These approaches aim to attenuate excessive inflammation, enhance tissue repair, or harness the antimicrobial potential of neutrophils.

### 8.2 Future directions and challenges

#### 8.2.1 Single‐cell technologies

The continued advancement of single‐cell technologies, including transcriptomics, proteomics, and metabolomics, will be crucial for further dissecting neutrophil heterogeneity and plasticity. Integrating these approaches with spatial transcriptomics and in vivo imaging will provide a more comprehensive understanding of neutrophil diversity and function within tissues.

#### 8.2.2 Tissue‐specific neutrophil biology

Further research is needed to elucidate the tissue‐specific factors that shape neutrophil phenotypes and functions. Understanding how different tissue microenvironments influence neutrophil behavior will be critical for developing targeted therapies that modulate neutrophil functions in specific organs or disease contexts.

#### 8.2.3 Neutrophil–microbiome interactions

The emerging role of neutrophils in shaping the microbiome and vice versa represents an exciting frontier. Unraveling the complex interplay between neutrophils, commensal microbes, and pathogens may lead to novel strategies for maintaining homeostasis and treating dysbiosis‐related disorders.

#### 8.2.4 Metabolic regulation of neutrophil function

Further investigation into the metabolic pathways that regulate neutrophil activation, survival, and effector functions may reveal new targets for therapeutic intervention. Understanding how metabolic reprogramming influences neutrophil plasticity could lead to metabolic‐based approaches for modulating neutrophil responses.

#### 8.2.5 Neutrophil memory and trained immunity

Exploring the concept of neutrophil memory and its role in trained immunity represents an intriguing area for future research. Elucidating the mechanisms underlying these phenomena could have significant implications for vaccine development and the treatment of chronic inflammatory diseases.

#### 8.2.6 Biomarkers and personalized medicine

Identifying reliable biomarkers that reflect neutrophil activation states and functional polarization will be crucial for patient stratification and personalized treatment approaches. Developing noninvasive methods to monitor neutrophil phenotypes in vivo remains a significant challenge.

#### 8.2.7 Therapeutic translation

While numerous neutrophil‐targeted therapies have shown promise in preclinical studies, their successful translation to the clinic faces several challenges. These include the potential for off‐target effects, the need for tissue‐specific targeting, and the complexity of modulating neutrophil functions without compromising host defense.

#### 8.2.8 Long‐term consequences of neutrophil modulation

As neutrophil‐targeted therapies advance, it will be crucial to assess the long‐term consequences of modulating neutrophil functions. This includes evaluating the potential impacts on immune memory, cancer surveillance, and overall immune homeostasis.

#### 8.2.9 Integrating neutrophil biology with systems immunology

Developing comprehensive models that integrate neutrophil biology with broader immune system dynamics will be essential for predicting the outcomes of neutrophil‐targeted interventions and understanding their systemic effects.

#### 8.2.10 Ethical considerations

As we develop more sophisticated tools for manipulating neutrophil functions, it will be important to address the ethical implications of these approaches, particularly in the context of enhancing human performance or modifying immune responses.

In conclusion, the field of neutrophil biology stands at an exciting crossroads, with the potential to revolutionize our approach to treating a wide range of diseases. By addressing these challenges and pursuing these future directions, we can harness the full therapeutic potential of neutrophils while deepening our understanding of their complex roles in health and disease. The journey ahead promises to yield transformative insights that will shape the future of immunology and medicine.

## AUTHOR CONTRIBUTIONS

W. F. H. and L. F. Y. wrote the manuscript. D. X. H. drew the figures. L. F. Y. and D. X. H. helped design the tables. W. F. H., Y. C. L., J. L. H., and G. X. L. helped design the manuscript structure. W. F. H., G. X. L., and Y. C. L. evaluated, reviewed, and revised the manuscript structure, ideas, and science. All authors have read and approved the final manuscript.

## CONFLICT OF INTEREST STATEMENT

Author Yih‐Cherng Liou is an Editorial board member of *MedComm*. Author Yih‐Cherng Liou was not involved in the journal's review of or decisions related to this manuscript. The other authors declared no conflicts of interest.

## ETHICS STATEMENT

Not applicable.

## Data Availability

Data availability is not applicable to this article as no new data were created or analyzed in this study.
